# Genome-wide association study and meta-analysis identify loci associated with ventricular and supraventricular ectopy

**DOI:** 10.1038/s41598-018-23843-z

**Published:** 2018-04-04

**Authors:** Melanie D. Napier, Nora Franceschini, Rahul Gondalia, James D. Stewart, Raúl Méndez-Giráldez, Colleen M. Sitlani, Amanda A. Seyerle, Heather M. Highland, Yun Li, Kirk C. Wilhelmsen, Song Yan, Qing Duan, Jeffrey Roach, Jie Yao, Xiuqing Guo, Kent D. Taylor, Susan R. Heckbert, Jerome I. Rotter, Kari E. North, Alexander P. Reiner, Zhu-Ming Zhang, Lesley F. Tinker, Duanping Liao, Cathy C. Laurie, Stephanie M. Gogarten, Henry J. Lin, Jennifer A. Brody, Traci M. Bartz, Bruce M. Psaty, Nona Sotoodehnia, Elsayed Z. Soliman, Christy L. Avery, Eric A. Whitsel

**Affiliations:** 1 Department of Epidemiology, Gillings School of Public Health, University of North Carolina, Chapel Hill, NC USA; 2 Carolina Population Center, University of North Carolina, Chapel Hill, NC USA; 3 Division of Cardiology, Department of Medicine, University of Washington, Seattle, WA USA; 4 Division of Epidemiology and Community Health, University of Minnesota, Minneapolis, MN USA; 5 Department of Genetics, University of North Carolina, Chapel Hill, NC USA; 6 Department of Biostatistics, University of North Carolina, Chapel Hill, NC USA; 7 Renaissance Computing Institute, University of North Carolina, Chapel Hill, NC USA; 8 Department of Computer Science, University of North Carolina, Chapel Hill, NC USA; 9 Research Computing Center, University of North Carolina, Chapel Hill, NC USA; 10 Institute for Translational Genomics and Population Sciences and Department of Pediatrics, Los Angeles Biomedical Research Institute at Harbor-UCLA Medical Center, Torrance, California USA; 11 Division of Genomic Outcomes, Department of Pediatrics, Harbor-UCLA Medical Center, Torrance, California USA; 12 Cardiovascular Health Research Unit and the Department of Epidemiology, University of Washington, Seattle, WA USA; 13 Division of Genomic Outcomes, Departments of Pediatrics and Medicine, Harbor-UCLA Medical Center, Torrance, California USA; 14 Carolina Center for Genome Sciences, University of North Carolina, Chapel Hill, NC USA; 15 Public Health Sciences Division, Fred Hutchinson Cancer Research Center, Seattle, WA USA; 16 Department of Epidemiology, University of Washington, Seattle, WA USA; 17 Epidemiological Cardiology Research Center, Department of Epidemiology and Prevention, Wake Forest University, Winston-Salem, NC USA; 18 Department of Public Health Sciences, Penn State University College of Medicine, Hershey, PA USA; 19 Department of Biostatistics, University of Washington, Seattle, WA USA; 20 Division of Medical Genetics, Department of Pediatrics, Harbor-UCLA Medical Center, Torrance, California USA; 21 Cardiovascular Health Research Unit, Departments of Biostatistics and Medicine, University of Washington, Seattle, WA USA; 22 Cardiovascular Health Research Unit, Departments of Medicine, Epidemiology, and Health Services, University of Washington, Seattle, WA USA; 23 Kaiser Permanente Washington Health Research Institute, Seattle, WA USA; 24 Department of Epidemiology, School of Medicine, Wake Forest University, Winston-Salem, NC USA; 25 Department of Medicine, University of North Carolina, Chapel Hill, NC USA

## Abstract

The genetic basis of supraventricular and ventricular ectopy (SVE, VE) remains largely uncharacterized, despite established genetic mechanisms of arrhythmogenesis. To identify novel genetic variants associated with SVE/VE in ancestrally diverse human populations, we conducted a genome-wide association study of electrocardiographically identified SVE and VE in five cohorts including approximately 43,000 participants of African, European and Hispanic/Latino ancestry. In thirteen ancestry-stratified subgroups, we tested multivariable-adjusted associations of SVE and VE with single nucleotide polymorphism (SNP) dosage. We combined subgroup-specific association estimates in inverse variance-weighted, fixed-effects and Bayesian meta-analyses. We also combined fixed-effects meta-analytic *t*-test statistics for SVE and VE in multi-trait SNP association analyses. No loci reached genome-wide significance in trans-ethnic meta-analyses. However, we found genome-wide significant SNPs intronic to an apoptosis-enhancing gene previously associated with QRS interval duration (*FAF1;* lead SNP rs7545860; effect allele frequency = 0.02; *P* = 2.0 × 10^−8^) in multi-trait analysis among European ancestry participants and near a locus encoding calcium-dependent glycoproteins (*DSC3;* lead SNP rs8086068; effect allele frequency = 0.17) in meta-analysis of SVE (*P* = 4.0 × 10^−8^) and multi-trait analysis (*P* = 2.9 × 10^−9^) among African ancestry participants. The novel findings suggest several mechanisms by which genetic variation may predispose to ectopy in humans and highlight the potential value of leveraging pleiotropy in future studies of ectopy-related phenotypes.

## Introduction

Supraventricular and ventricular ectopy (SVE, VE) are extra, abnormal depolarizations at non-sinus atrial, atrioventricular, or ventricular foci. The electrocardiographic hallmarks of SVE include absent or morphologically distinct P waves or PR intervals of different duration^1,2^, while those of VE include widened, morphologically bizarre single or multiple QRS complexes not preceded by P waves^1,2^.

As defined above, SVE and VE are common, but often occur as intermittent, asymptomatic, and/or clinically isolated events that increase in frequency with age^3,4^ depending on the method and duration of observation. On resting, supine, ten-second, standard twelve-lead electrocardiograms (ECGs), the prevalence of isolated SVE (<1%)^5^ and VE (~1%)^6^ is low, but higher in those with diseases of the heart^3^, lung^7^, brain^8^, kidney^9^, and/or exposure to medications used to treat them^6^. Moreover, SVE has been associated with ischemic heart disease mortality in persons free of such diseases^10^ and can trigger e.g. atrial fibrillation^11^. VE also is associated with ventricular fibrillation and sudden cardiac death^12,13^. Precipitants of SVE and VE are therefore of great clinical and public health interest.

Although behavioral and environmental precipitants (stress, tobacco, alcohol, caffeine, air pollution, exercise)^6,14–16^ have been studied, genetic predisposition to and heritability of SVE and VE in humans have not. Genome-wide association studies (GWAS) have nonetheless illuminated a shared genetic architecture at e.g. the *SCN5QA/10A* locus and distinct pathophysiological mechanisms underlying QT, PR, and QRS durations and atrial fibrillation^17^. Similarly, multi-trait analyses have identified novel, mechanistically important loci previously undetected by single-trait analyses^18^. Given established genetic mechanisms of arrhythmogenesis^19^ and familial aggregation of other supraventricular^20^ and ventricular^21^ arrhythmias, we performed the first GWAS examining the hypothesis that genetic variation on a genome-wide scale influences electrocardiographic manifestation of SVE and VE in a large study of diverse ancestries.

## Methods

### Cohorts and participants

We studied SVE and VE among 42,976 and 44,131 participants who provided written, informed consent to use their genetic data. The participants were of European, African, or Hispanic/Latino ancestry and originated in five large, prospective cohorts: the Atherosclerosis Risk in Communities (ARIC) study^22^, the Women’s Health Initiative Clinical Trial (WHI)^23^, the Multi-Ethnic Study of Atherosclerosis (MESA)^24^, the Cardiovascular Health Study (CHS)^25^, and the Hispanic Community Health Study/Study of Latinos (HCHS/SOL)^26^. We excluded first-degree relatives, and participants with low quality ECGs, pacemakers, or anti-arrhythmic medication use at each visit. Institutional Review Boards (IRB) at participating institutions approved all cohort-specific study protocols.

Briefly, ARIC is a prospective, longitudinal study of cardiovascular disease. Between 1987 and 1989, the study enrolled a probability sample of 15,792 men and women of African American and European descent aged 45–64 years^22^ from four US communities: Forsyth County, NC; Jackson, MS; suburban Minneapolis, MN; and Washington County, MD. Cohort members completed five visits: (1) 1987–1989; (2) 1990–1992; (3) 1993–1995; (4) 1996–1999; and (5) 2011–2013, at which ECGs were recorded.

WHI is randomized, controlled trial of hormone (estrogen +/− progestin) therapy, calcium/vitamin D supplementation, and dietary modification on the risk of breast and colorectal cancer, cardiovascular disease, and bone fracture^23,27^. A total of 68,132 postmenopausal women aged 50–79 years old were enrolled from 40 clinical centers in the U.S. between 1993 and 1998. Eligible women were followed-up at years 1, 3, 6 and 9, during which ECGs were recorded. For the present study, we included European ancestry participants from three sub-cohorts: GARNET (Genomics and Randomized Trials Network), MOPMAP (Modification of Particulate-Matter-mediated Arrhythmogenesis in Populations), and WHIMS (Women’s Health Initiative Memory Study). We included African and Hispanic/Latino ancestry participants from the SNP Health Association Resource (SHARe) project. Sub-cohorts are described in the Supplementary Methods.

MESA is a cohort study of subclinical cardiovascular disease and the risk factors that predict progression to clinically overt disease^24^. The 6,814 MESA participants were asymptomatic men and women aged 45–84 [38% European ancestry; 28% African ancestry; 22% Hispanic/Latino; and 12% Asian (mainly Chinese) ancestry]. During 2000–2002, participants were recruited from six US communities: (Forsyth County, NC; New York, NY; Baltimore, MD; St. Paul, MN; Chicago, IL, and Los Angeles County, CA). After an initial physical examination, there were four additional examination periods (17–20 months long). ECGs were recorded at exams 1 and 5 (2010–2011), but herein, we only use those from the former.

CHS is a cohort study of risk factors for coronary heart disease and stroke in adults ≥65 years conducted across four field centers: Sacramento County, CA; Washington County, MD; Forsyth County, NC; and Pittsburgh, PA^25^. The original, predominantly European ancestry cohort of 5,201 persons was recruited in 1989–1990 from random samples of Medicare eligibility lists; subsequently, an additional predominantly-African-American cohort of 687 persons were enrolled for a total sample of 5,888. ECGs were recorded at annual visits, but herein, we only used those from baseline.

HCHS/SOL is a study focused on describing the prevalence of risk and protective factors for chronic conditions, and to quantify all-cause mortality, fatal and non-fatal cardiovascular disease and pulmonary disease, and pulmonary disease exacerbation over time^26^. From 2008–2011, 16,415 Hispanic/Latino individuals aged 18–74 were recruited from randomly selected households in four US communities: Bronx, NY; Chicago, IL; Miami, FL; and San Diego, CA using a stratified two-stage area probability sampling design. HCHS/SOL includes participants who self-identified as having Hispanic/Latino background, the largest groups being Central American (n = 1,730), Cuban (n = 2,348), Dominican (n = 1,460), Mexican (n = 6,471), Puerto-Rican (n = 2,728), and South American (n = 1,068). At the time of the present study, participants only had an ECG at the baseline visit.

### Electrocardiography

Trained, certified technicians digitally recorded ECGs at visits 1–5 in ARIC^28^; screening and annual visits 3, 6, and 9 in WHI^29^; and, for this analysis, the baseline visit in MESA^24^, CHS^25^, and HCHS/SOL^26^. Technicians used comparable procedures for preparing participants, placing electrodes, recording ECGs with MAC PC electrocardiographs (GE Marquette Electronics, Inc., Milwaukee, WI), and telephonically transmitting them to the Epidemiological Cardiology Research Center (Wake Forest School of Medicine, Winston Salem, NC) for inspection, identification of technical errors/inadequate quality, and analysis using the Marquette 12-SL program (2001 version, GE Marquette, Milwaukee, WI).

### Identification of Supraventricular and Ventricular Ectopy

Since SVE and VE often occur intermittently and in isolation, presence of each phenotype on the ECG was determined independently at each visit. Supraventricular and ventricular ectopic beats were separately detected by computer algorithms based on the Minnesota Code (MC) and visually over-read by physicians (ARIC, WHI, MESA, HCHS/SOL). SVE was defined as ≥1 supraventricular ectopic beat (MC8.1.1, 8.1.3–8.1.5) and VE as ≥1 ventricular ectopic beat (MC8.1.2–8.1.3, 8.1.5) during the ten-second recording^1^. Because few participants had ≥1 ectopic beat at a given visit, we analyzed SVE and VE as binary variables (0: absence, ≥1: presence).

### Genotyping, Quality Control, and Imputation

Each cohort or study performed genome-wide genotyping using Affymetrix or Illumina arrays and used similar quality control thresholds for excluding SNPs and samples (Supplementary Table S1). Genotypes were imputed using HapMap 2, HapMap 2 and 3, or 1000 Genomes Phase 1 (version 3, March 2012 release) reference panels. To enable cross-platform comparisons, Build 36 coordinates were converted to Build 37, and analyses were restricted to SNPs present in HapMap 2.

### Statistical analysis

We stratified cohort participants by ancestry (and study) into thirteen subgroups of European (ARIC, CHS, MESA, WHI-GARNET, WHI-MOPMAP, WHI-WHIMS), African (ARIC, CHS, MESA, WHI-SHARe), and Hispanic/Latino (MESA, HCHS/SOL, WHI-SHARe) descent. For each of the thirteen ancestry-stratified subgroups, GWA analyses followed a standard protocol leveraging the availability of repeat ECGs, when available, to increase power. In cohorts with multiple ECGs per participant over time (ARIC, WHI), we estimated ectopy-SNP associations using generalized estimating equation (GEE) methods^30^, a logit link, and an exchangeable working correlation structure to control for correlation of repeated measures (R geepack package). In studies with one ECG per participant (MESA, CHS), we estimated associations using logistic regression (SNPTEST, R geeglm package). Though multiple ECGs were available in MESA and CHS, only baseline visit data were used in accordance with analytic pipelines. In HCHS/SOL, we estimated associations among unrelated (at the 3^rd^ degree level) participants (one per household) using a generalized linear model and a Firth test^31^ to account for small numbers of cases (R logistf package), assuming Census block group effects were negligible. We adjusted all models for age (year), sex (studies containing >1), season (quarter), study center (ARIC, CHS, MESA, HCHS/SOL) or geographic region (WHI), and ancestry principal components estimated using Eigenstrat^32^ (ARIC, CHS, MESA, WHI) or PC-AiR^33^ (HCHS/SOL).

Within subgroups, we compared observed *P*-values for each SNP with expected values from a χ^2^ distribution using quantile-quantile (Q-Q) plots and genomic inflation factors (lambda). To eliminate statistical artifacts at low allele and ectopy frequencies, the comparisons excluded SNPs with an effective number of minor alleles present in exposed participants (defined as 2 × number of exposed participants × minor allele frequency × imputation quality) <10 or a log odds ratio >10. After filtering, thirteen and twelve subgroups contributed to the SVE and VE meta-analyses (MESA Hispanic/Latinos did not meet filtering thresholds due to infrequency of VE; n = 17).

### Meta-analysis

We prioritized trans-ethnic analyses to maximize power and generalizability, given previous research suggesting that causal variants are typically relevant across populations^34^, but also conducted ancestry-specific analyses given the potential for differences in linkage disequilibrium (LD) and allele frequency among populations. Analyses involved combining subgroup- and ancestry-specific summary results in 1) fixed-effects, inverse-variance-weighted meta-analyses with genomic control (METAL) and 2) trans-ethnic Bayesian meta-analysis (MANTRA)^35^ to account for allelic heterogeneity among ancestry groups. MANTRA clustered similar populations according to allele frequencies, allowed for varying allele effects across populations, and produced Bayes’ factors (BFs) for each ectopy-SNP association and its posterior probability of heterogeneity (*P*_het_). We also performed multi-trait SNP association analyses that combined *t*-test statistics from fixed-effects meta-analyses of SVE and VE, using adaptive sum of powered score (aSPU) methods to investigate potential pleiotropy^36^. While etiologies of SVE and VE may differ, combination was justified by extant knowledge of their shared precipitants^14,15^, potential co-occurrence (MC 8.1.3 or 8.1.5)^1^, and difficulty distinguishing them from each other^37^. Multi-trait analyses provided *P*-values for genetic correlations among traits, but no effect estimates.

By convention, we set genome-wide significance at *P* < 5.0 × 10^−8^ and suggestive significance at *P* < 2.5 × 10^−6^ for fixed-effects meta-analyses. For Bayesian meta-analyses, we used a log_10_BF ≥6.0 as a genome-wide threshold for discovery (to approximate the performance of a *P* < 5.0 × 10^−8^)^38^, a *P*_het_ < 0.5 as a liberal indicator of homogeneity among subgroups, and ≥ two contributing racial/ethnic groups as a threshold for performing meta-analysis. Suggestive SNPs had log_10_BF ≥5.0, *P*_het_ <0.5, and ≥ two contributing racial/ethnic groups. We report sub-threshold hits for trans-ethnic meta-analyses because they had the largest number of participants. We considered SNPs with ancestry-specific LD *r*^2^ < 0.2 as independent. We summarized results from genomically controlled meta-analyses in Q-Q plots, Manhattan plots of the -log_10_*P* value versus SNP position, and regional association plots. We functionally annotated lead and correlated SNPs (*r*^2^ ≥ 0.8) in relevant cardiac tissues using HaploReg v4.1^39^ and visualized relevant tracks using the UCSC Genome Browser. We estimated heritability in European ancestry populations (ARIC, WHI-MOPMAP) using Genome-wide Complex Trait Analysis^40^.

### Data availability

Complete results are available on dbGAP at https://www.ncbi.nlm.nih.gov/projects/gap/cgi-bin/study.cgi?study_id=phs000930.v5.p1. Primary data are available from the parent studies conditional on review and approval of requests by cohort-specific presentation and publication committees.

## Results

### Study characteristics

A total of 42,976 participants in thirteen subgroups contributed to the SVE analysis, of whom 22% were of African ancestry, 26% Hispanic/Latino, and 76% female (Table 1). On average, these participants were aged 66.3 years and contributed 2.2 visits (range:1–5), at which 2–10% of them had SVE at one or more visits. Estimated heritability (standard error(SE)) of SVE in ARIC was 3.2% (3.4%). A total of 44,131 participants in twelve subgroups contributed to the VE analysis, of whom 21% were of African ancestry, 25% Hispanic/Latino, and 74% female. On average, these participants were aged 67.7 years and also contributed 2.2 visits, during which 1–8% had VE at one or more visits, except in MOPMAP, which sampled VE cases and controls in equal proportions. Baseline prevalence of VE was <3% in all subgroups, except in MOPMAP. Heritability of VE in ARIC and WHI-MOPMAP were 9.4% (3.4%) and 32% (14%). Lambdas from subgroup-specific Q-Q plots of SVE and VE ranged from 0.99 to 1.04 (Supplementary Figs S1 and S2).

**Table 1:** Characteristics of the subgroups, by ancestry.

Supraventricular ectopy						Ventricular ectopy				
Ancestry	n	Female	Age, yr	Visits	SVE at any visit	n	Female	Age, yr	Visits	VE at any visit
		n (%)	mean (SD)^†^	mean (SD)	n (%)		n (%)	mean (SD)^†^	mean (SD)	n (%)
European	**22,517**					**23,672**				
ARIC	9,055	4,802 (53.0)	60.2 (8.3)	4.0 (1.0)	730 (8.1)	9,055	4,802 (53.0)	60.2 (8.2)	4.0 (1.0)	714 (7.9)
CHS	3,081	1,901 (61.7)	72.2 (5.3)	1.0 (0)	102 (3.3)	3,081	1,901 (61.7)	72.2 (5.3)	1.0 (0)	135 (4.4)
MESA	2,491	1,302 (52.3)	62.7 (10.2)	1.0 (0)	87 (3.5)	2,491	1,302 (52.3)	62.7 (10.2)	1.0 (0)	52 (2.1)
WHI-GARNET	2,091	2,091 (100)	66.5 (6.8)	2.6 (0.8)	160 (7.6)	1,944	1,944 (100)	69.2 (7.2)	2.6 (0.9)	41 (2.1)
WHI-MOPMAP	1,458^‡^	1,458 (100)	64.1 (6.6)	2.8 (0.7)	103 (7.1)	2,957	2,957 (100)	68.5 (7.2)	3.0 (0.7)	1,475 (49.9)
WHI-WHIMS	4,341	4,341 (100)	70.8 (3.8)	2.5 (0.7)	436 (10.0)	4,144	4,144 (100)	73.6 (4.5)	2.5 (0.7)	51 (1.2)
African	**9,274**					**9,274**				
ARIC	2,381	1,495 (62.8)	58.7 (8.1)	3.6 (1.1)	224 (9.4)	2,381	1,495 (62.8)	58.7 (8.1)	3.6 (1.1)	179 (7.5)
CHS	767	487 (63.5)	72.8 (5.7)	1.0 (0)	55 (7.2)	767	487 (63.5)	72.8 (5.7)	1.0 (0)	58 (7.6)
MESA	1,600	859 (53.7)	62.2 (10.1)	1.0 (0)	72 (4.5)	1,600	859 (53.7)	62.2 (10.1)	1.0 (0)	33 (2.1)
WHI-SHARe	4,526	4,526 (100)	61.9 (6.8)	2.6 (0.8)	301 (6.6)	4,526	4,526 (100)	64.9 (7.2)	2.6 (0.8)	211 (4.7)
Hispanic/Latino	**11,185**					**11,185**				
HCHS/SOL	7,909	5,727 (27.4)	47.3 (13.3)	1.0 (0)	263 (2.1)	7,909	5,728 (27.4)	47.3 (13.2)	1.0 (0)	94 (0.7)
MESA	1,440	743 (51.6)	61.4 (10.2)	1.0 (0)	30 (2.1)	1,440§	743 (51.6)	61.4 (10.2)	1.0 (0)	16 (1.1)
WHI-SHARe	1,836	1,836 (100)	60.6 (6.4)	2.6 (0.8)	61 (3.3)	1,836	1,836 (100)	63.7 (6.8)	2.6 (0.8)	58 (3.2)
***Total***	***42,976***	***76%***	***66.3***	***2.2***	***2.1–10.0%***	***44,131***	***74%***	***67.7***	***2.2***	***0.7–49.9%***

ARIC = Atherosclerosis Risk in Communities study; CHS = Cardiovascular Health Study; GARNET = Genome-wide Association Research Network into Effects of Treatment; HCHS/SOL = Hispanic Community Health Study/Study of Latinos; MESA = Multi-Ethnic Study of Atherosclerosis; MOPMAP = Modification of PM-Mediated Arrhythmogenesis in Populations; n = number; SD = standard deviation; SHARe = SNP Health Association Resource; SVE = supraventricular ectopy; VE = ventricular ectopy; WHI = Women’s Health Initiative; WHIMS = Women’s Health Initiative Memory Study.

^†^For studies with multiple visits, mean age is the age on date of ECG computed across all visits.

^‡^Controls only in the trans-ethnic meta-analysis of SVE.

^§^After filtering on expected heterozygosity, MESA Hispanics did not contribute to the trans-ethnic meta-analysis of VE.

### Trans-ethnic meta-analyses

No SNP associations exceeded a genome-wide threshold for SVE or VE in trans-ethnic, fixed-effects meta-analyses; however, sub-threshold associations were identified for both phenotypes (Supplementary Table S2). Furthermore, Bayesian and multi-trait analyses (not shown) did not identify trans-ethnic loci (Supplementary Fig. S3).

### Ancestry-specific meta-analyses: European

**Figure 1:**
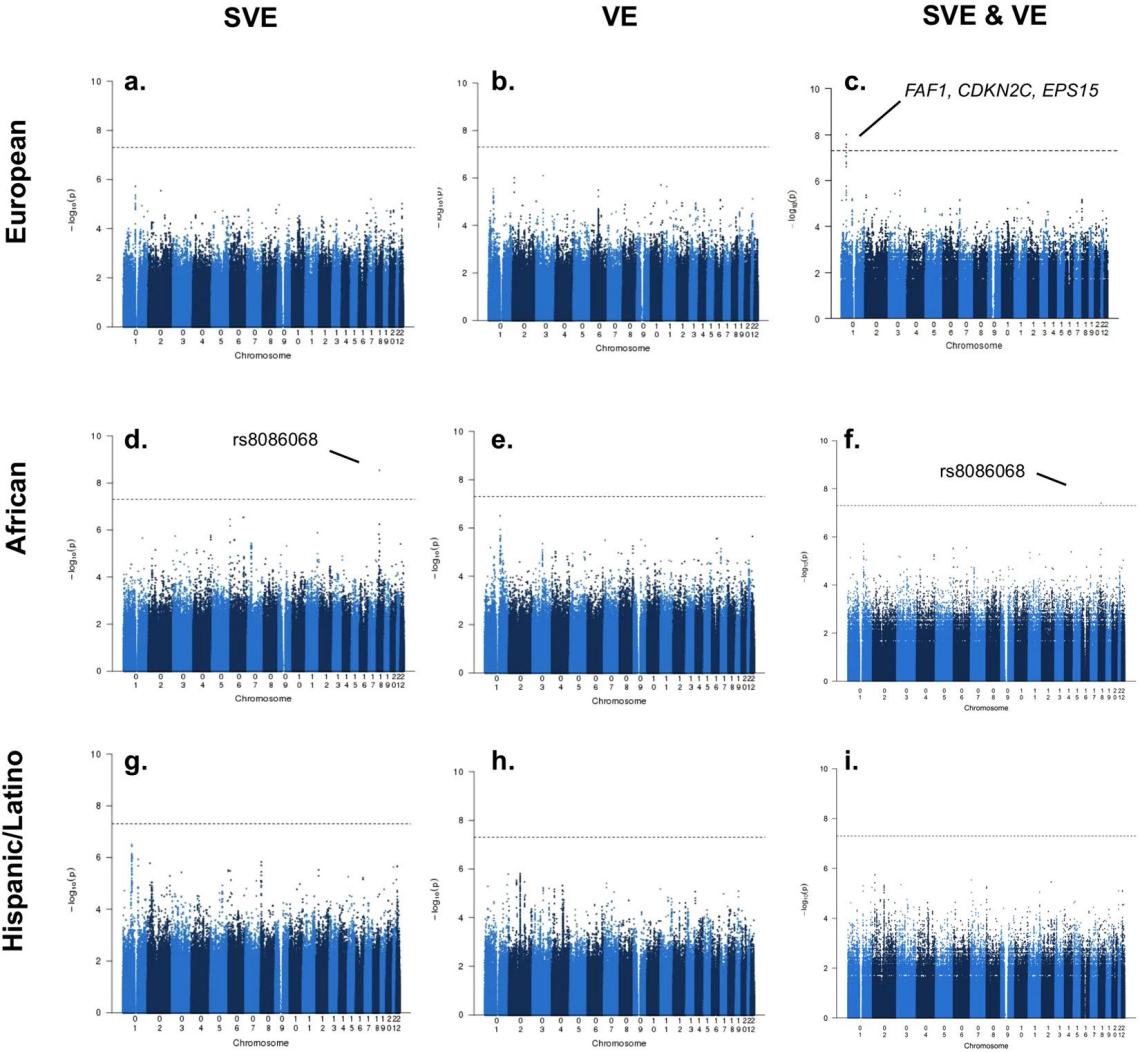
Manhattan plots of *P-*values from ancestry-specific fixed-effects meta-analyses of SVE and VE, and multi-trait analysis of SVE & VE. (Column 1, Panels a,d and g) Ancestry-specific fixed-effects meta-analyses of SVE; (Column 2, Panels b,e and h) Ancestry-specific fixed-effects meta-analyses of VE; (Column 3, Panels c,f and i) Multi-trait analysis of SVE & VE. Dotted horizontal line indicates the genome-wide significance threshold (5 × 10^−8^).

There were no genome-wide significant associations in fixed-effects meta-analyses of European ancestry studies of SVE or VE. However, multi-trait analysis identified a locus on chromosome 1 jointly associated with SVE and VE (*P* = 2.0 × 10^−8^; Panels A,B in Fig. 1). The lead SNP at this locus, rs7545860 (effect allele frequency [EAF] = 0.02), is an intron variant in *Fas Associated Factor 1* (*FAF1*) and 92 kb 5′ from *Cyclin-Dependent Kinase Inhibitor 2C* (*CDKN2C*) (Table 2; Panel C in Fig. 1; Supplementary Fig. S4). Rs7545860, and correlated SNPs (*r*^2^ ≥ 0.2) including rs72692218 and rs66462949, reside in a genomic region including deoxyribonuclease (DNase I) hypersensitive sites, regulatory motifs, and putative enhancer/promoter histone signals in fetal heart, right atrium and ventricle, and/or aorta (Supplementary Fig. S5). This locus may also include the epidermal growth factor receptor pathway substrate 15 (*EPS15*) gene through SNPs in LD with rs7545860 (rs17106627 and rs12022046) (Supplementary Fig. S6). These SNPs are also in regions containing DNase I hypersensitivity sites, DNA methylation sites, putative enhancer/promoter histone marks, and regulatory motifs in cardiomyocytes and cardiac fibroblasts (Supplementary Fig. S7).

**Table 2:** SNPs with genome-wide significant (*P* < 5 × 10^−8^) associations in ancestry-specific multi-trait analysis of SVE & VE.

**Ancestry**	**SNP**	**Genomic Region (Build 37)**	**Gene**	**Alleles** ^**†**^	**Effect Allele Frequency**	***P-value***		
						**SVE** ^§^	**VE**	**SVE & VE**
European	rs7545860^**‡**^	C1:51341666	*FAF1,CDKN2C*	A/G	0.98	1.0 × 10^−4^	2.8 × 10^−6^	1.0 × 10^−8^
African	rs8086068	C18:28363623	—	A/C	0.17	2.9 × 10^−9^	8.3 × 10^−1^	4.0 × 10^−8^

C = chromosome; SNP = single nucleotide polymorphism; SVE = supraventricular ectopy; VE = ventricular ectopy.

^**†**^Effect/other.

^‡^Associations with two SNPs in LD with rs7545860 (rs17106627*[EPS15]* and rs12022046[intergenic]) also were significant.

^§^Direction in European ancestry = --???? (ARIC – WHI-WHIMS – WHI-MOPMAP controls – WHI-GARNET controls – CHS – MESA); Direction in African ancestry = ----(ARIC – WHI-SHARE – CHS – MESA).

^¶^Direction in European ancestry = --???? (ARIC – WHI-MOPMAP – WHIMS –WHI-GARNET controls – MESA – CHS); Direction in African ancestry = + -?- (ARIC – WHI-SHARE – MESA – CHS).

### Ancestry-specific meta-analyses: African

Among African ancestry participants, fixed-effects meta-analysis of SVE identified a novel signal on chromosome 18 (lead SNP rs8086068; EAF = 0.17; *P* = 2.87 × 10^−9^), associated with a 75% increased odds of SVE per copy of the C allele (95% CI: 1.46–2.11) (Panel D in Fig. 1). This variant also was directionally consistent, if not significant, among European ancestry studies and one Hispanic/Latino ancestry subgroup (Supplementary Fig. S8). Multi-trait analyses identified this same lead SNP (*P* = 4.0 × 10^−8^), driven by its association with SVE (Table 2; Panel F in Fig. 1). While intergenic, rs8086068 is 206 kb 3′ from the desmocolin 3 (*DSC3*) gene, one of a family of desmocolin genes clustered in the area, though the SNP is separated from the gene family by a recombination spike and may not interact with it (Supplementary Figs S8, S9). Functional annotation indicates that three SNPs in LD (*r*^2^ ≥ 0.2) with this lead SNP using the 1000G AFR referent population (rs2097047, rs17711533, rs17711559) occur within DNase I hypersensitivity sites in fetal heart tissue (Supplementary Fig. S10). No SNPs met the genome-wide threshold for significance among African ancestry studies in fixed-effects meta-analyses of VE (Panel E in Fig. 1).

### Ancestry-specific meta-analyses: Hispanic/Latino

No SNPs met the genome-wide threshold among Hispanic/Latino ancestry studies in fixed-effects meta-analyses of SVE or VE, or in multi-trait meta-analyses (Panels G-I in Fig. 1). Complete results are available on dbGAP at https://www.ncbi.nlm.nih.gov/projects/gap/cgi-bin/study.cgi?study_id=phs000930.v5.p1.

## Discussion

This first GWAS of ectopy identified two biologically plausible loci among European and African ancestry individuals. It identified the *FAF1/CDKN2C/EPS15* locus (chromosome 1) in multi-trait meta-analyses of SVE and VE among European ancestry individuals. Earlier GWAS have associated this locus with QRS duration^41^. It also identified a second locus among African ancestry individuals, approximately 206 kb 3′ from a desmocolin gene cluster that includes *DSC3* and *DSC2*, the latter previously associated with arrhythmogenic cardiomyopathy (ACM)^42^. Together, these findings provide insight into putative mechanisms underlying genetic susceptibility to ectopy.

Contrary to expectation, this GWAS of ectopy did not identify any loci meeting the genome-wide threshold for significance in trans-ethnic, fixed-effects or Bayesian meta-analyses of either phenotype. Restriction of analyses to HapMap 2 SNPs may be one reason why none were identified, given the admittedly limited genomic coverage of this reference panel, although restriction also enabled cross-platform comparisons. Heterogeneity of association among races/ethnicities due to differences in imputation quality or minor allele frequency may be another. Lastly, as large as our study is, an even larger study may be required to adequately power the identification of trans-ethnically important variants, as is further discussed, below.

The European ancestry locus identified by rs7545860 is intronic to *FAF1*, an apoptosis protein-encoding gene previously implicated in GWAS of QRS duration^43^. Two SNPs in LD with rs7545860 (rs72692218; rs66462949) are intronic to the nearby gene *CDKN2C*, a cyclin-dependent kinase inhibitor dually implicated by that GWAS. The lead SNP is also in LD with rs17391905, a *FAF1* and *CDKN2C* SNP identified by Sotoodehnia *et al*. (*r*^2^ = 0.53, multi-trait aSPU *P* = 1.60 × 10^−7^). Additional SNPs in LD with rs7545860 include intronic variants (rs17106627, rs12022046) of *EPS15*, a gene that encodes a calcium-binding protein involved in receptor-mediated endocytosis of epidermal growth factor, but has no previously established role in arrhythmogenesis. Functional annotation for these SNPs suggests potential involvement with histone modification and enhancer activity in fetal heart.

It is notable that the aforementioned European ancestry locus (*FAF1/CDKN2C/EPS15*) was only identified when using adaptive sum of powered score methods to investigate pleiotropy. This finding highlights the potential value of leveraging pleiotropic effects in future studies of ectopy-related phenotypes. Indeed, examining them may well improve understanding of biological mechanisms underlying correlated traits.

No GWAS has been published to date relating arrhythmia to genetic variation in desmocolin cluster genes, including *DSC3*. The desmocolin gene cluster is of interest because the desmocolins are calcium-dependent glycoproteins involved in cardiac intercellular connections and neighboring gene *DSC2* is associated with ACM, a congenital heart disorder characterized by right ventricular fibrofatty infiltration, myocardiocyte apoptosis, gap junction pathophysiology, supraventricular/ventricular arrhythmias, and sudden cardiac death^42^. Moreover, several SNPs in LD with lead SNP rs8086068 are located within DNase I hypersensitivity sites in fetal heart tissue, suggesting potential involvement in tissue-specific regulation. We also demonstrated that this variant was directionally consistent among European ancestry studies and one Hispanic/Latino ancestry subgroup, suggesting that differences in risk factors or allelic effects among races/ethnicities may explain the ancestral heterogeneity of effects, a possibility deserving further study. Several other loci that reached the threshold for suggestive significance in trans-ethnic meta-analyses also have biologically plausible relationships with ectopy (Supplementary Discussion).

In addition to the loci discussed above, this paper adds to the literature an estimate of heritability for SVE and VE in European ancestry populations. In lieu of available family-based data, we estimated heritability using two cohorts with the largest number of ectopy cases (ARIC; WHI-MOPMAP) and among European ancestry participants because of the difficulty obtaining minority reference populations. Our finding that the estimated heritability of VE, a binary phenotype, differed in ARIC (9.4%, SE = 3.4%) and WHI-MOPMAP (32%, SE = 14%) is partly attributable to the difference in VE prevalence (and design) between those populations^44,45^. In our study, WHI-MOPMAP sampled VE cases and controls in equal proportions (i.e. 50% of participants had ectopy), but among ARIC European ancestry participants, the prevalence of VE was only 7.9%. The SVE heritability estimate was likely influenced by the same factors. Although the generalizability of such estimates outside of ARIC and WHI-MOPMAP is unknown and the estimates are not directly comparable to those estimated from pedigree data^44,45^, they remain notable findings from this study.

This work has several limitations that deserve consideration. Despite many participants in this meta-analysis, low prevalence of ectopy as measured by brief ECGs and limited genomic coverage of HapMap 2—especially in non-European ancestry populations—limited its overall power to identify trans-ethnic signals. By extension, our ability to detect ancestry-specific signals also was limited. Modest power is a well-known limitation of GWAS involving small populations, cross-sectional designs, infrequent outcomes, and brief ECG recordings. However, ectopy has not been examined in a multi-ethnic GWAS, so to examine it, we leveraged the following: (1) imputed genomic data from five cohorts including multiple ancillary studies and thirteen ancestry-stratified subgroups collectively representing >42,000 participants; (2) ECG data from up to five recordings per participant and eleven years of follow-up; (3) relatively powerful, longitudinal and meta-analytic methods that exploit ancestral heterogeneity^35^; and (4) multi-trait SNP association methods that exploit phenotypic correlation^36^. Leveraging multi-ethnic cohorts and these analytical methods powered the discovery and localization of ectopy-SNP associations, albeit based on ten-second ECG recordings.

We acknowledge that longer ECG recording durations are essential for detecting ectopy with sensitivity. Although the relative sensitivity of short ECG recordings for ectopy is low—even when repeated—paroxysmal arrhythmias frequent enough to be captured by insensitive, but highly specific, short recordings may have more prognostic significance than those so infrequent that they require long recordings to capture them^15^. Moreover, the bias of odds ratios reported here approaches zero because specificity of physician-verified ECGs for ectopy approaches 100%^46^ while their sensitivity among participants with and without a given variant is identical^47^. It is also possible that short ECG recordings capture frequent ectopy known to increase the risk of myocardial infarction, cardiac, and all-cause mortality *in addition to* infrequent ectopy associated with a relatively benign prognosis. The group to whom inferences can be made may therefore be heterogeneous. Finally, because independent replication was not feasible due to the current paucity of genotyped cohorts with physician-verified ectopy, we accordingly acknowledge that findings may be due to chance. These considerations underscore the need for further confirmation of our findings.

## Conclusions

Given these limitations, we view the study findings as hypothesis-generating and have provided publicly accessible summary statistics from ancestry-specific fixed-effects meta-analyses on dbGAP to facilitate external replication. But under those hypotheses, we also provide evidence that variants in *FAF1*/*CDKN2C, EPS15, DSC2/3*, and *SCN5A* on chromosomes 1, 3, and 18 contribute to the genetic risk of supraventricular/ventricular ectopy and arrhythmogenesis in humans via plausible cellular, intercellular, and cationic mechanisms involving myocardiocyte apoptosis, desmosome-related gap junction abnormality, sodium channelopathy, and electrocardiographically manifest derangement of normal atrioventricular physiology.

## Electronic supplementary material

Supplementary Information
