# Supplementary material for: Genome-wide association study and meta-analysis identify loci associated with ventricular and supraventricular ectopy
Source: Sci Rep. 2018 Apr 4;8:5675. doi: 10.1038/s41598-018-23843-z (PMC5884864; doi:10.1038/s41598-018-23843-z)
Supplement: Supplementary file 1 — Supplementary Information [file 41598_2018_23843_MOESM1_ESM.pdf]

Genome-wide association study and meta-analysis identify loci associated with ventricular and supraventricular ectopy

Melanie D. Napier*; Nora Franceschini; Rahul Gondalia; James D. Stewart; Raúl Méndez-Giráldez; Colleen M. Sitlani; Amanda A. Seyerle; Heather M. Highland; Yun Li, Kirk C. Wilhelmsen; Song Yan; Qing Duan; Jeffrey Roach; Jie Yao; Xiuqing Guo; Kent D. Taylor; Susan R. Heckbert; Jerome I. Rotter; Kari E. North; Alexander P. Reiner; Zhu-Ming Zhang; Lesley F. Tinker; Duanping Liao; Cathy C. Laurie; Stephanie M. Gogarten; Henry J. Lin; Jennifer A. Brody; Traci M. Bartz; Bruce M. Psaty; Nona Sotoodehnia; Elsayed Z. Soliman; Christy L. Avery; Eric A. Whitsel*

**Correspondence to mdnapier@email.unc.edu or eric_whitsel@med.unc.edu*

Contents

Supplementary Methods: Description of WHI Sub-cohorts **3**

Supplementary Discussion of Suggestive Findings **4**

Supplementary Table S1. Genotyping, quality control, imputation & analysis, by cohort and study **5**

Supplementary Table S2. SNPs with suggestive ($5.0 \times 10^{-8} < P < 2.5 \times 10^{-6}$) evidence of association with SVE and VE after fixed-effects meta-analysis of European, African, and Hispanic/Latino ancestry groups **7**

Supplementary Figure S1. Quantile-quantile plots for each study that contributed to meta-analysis of supraventricular ectopy. **8**

Supplementary Figure S2. Quantile-quantile plots for each study that contributed to meta-analysis of ventricular ectopy. **9**

Supplementary Figure S3. Quantile-quantile and Manhattan plots from trans-ethnic, fixed-effects meta-analysis of (A) supraventricular ectopy (SVE) and (B) ventricular ectopy (VE); and from trans-ethnic, multi-trait analysis of (C) SVE & VE. **10**

Supplementary Figure S4. Regional plot (A) of rs7545860 from multi-trait analysis of SVE & VE among European ancestry populations, and forest plots of rs7545860 from fixed effects analysis of SVE (B) & VE (C) among European ancestry populations. **11**

Supplementary Figure S5. UCSC Genome Brower displaying rs7545860 (highlighted), SNPs in linkage disequilibrium ($r2 \geq 0.2$ using EUR reference), and tracks representing DNAse1 hypersensitivity and DNA methylation in cardiac tissues. **12**

Supplementary Figure S6. (A) Hi-C data on 3D spatial contacts show that rs7545860 and rs17106627, rs12022046 belong to possibly different topological associated domains (TAD). 13

Supplementary Figure S7. UCSC Genome Brower displaying rs17106627 (highlighted) and tracks representing DNAse1 hypersensitivity and DNA methylation in cardiac tissues. 14

Supplementary Figure S8. Regional (A) and forest plots (B) of the association of rs8086068 and *supraventricular ectopy* in European, African, and Hispanic/Latino ancestry cohorts. 15

Supplementary Figure S9. (A) Hi-C data on 3D spatial contacts show that rs8086068 may be within the same topological associated domains (TAD) with *DSC3*. (B) In contrast, virtual 4-D figure centered at rs8086068 show little interaction with *DSC3*. 16

Supplementary Figure S10. UCSC Genome Brower displaying rs8086068 (highlighted), SNPs in linkage disequilibrium ($r2 \geq 0.2$ using AFR reference), and tracks representing DNAse1 hypersensitivity and DNA methylation in cardiac tissues. 17

Study-specific acknowledgements and funding sources for participating studies 18

References 20

Supplementary Methods: Description of WHI Sub-cohorts

For the present study, WHI women of European ancestry were clinical trial (CT) participants recruited from three sub-cohorts: GARNET (Genomics and Randomized Trials Network), MOPMAP (Modification of Particulate-Matter-mediated Arrhythmogenesis in Populations), and WHIMS (Women's Health Initiative Memory Study). GARNET[1] is a nested case-control sub-study of 497 coronary heart disease, 342 stroke, 304 venous thromboembolism, and 1,043 incident diabetes cases from the parent WHI hormone therapy trial, 167 cases of more than one type, and 2,344 matched controls free of all four case conditions by the end of the trial. MOPMAP[2] is a nested case-control cohort of 1,537 white women who developed VE at least once during all their visits (cases) and a stratified, random sample of 1,535 controls from the same clinical centers, calendar years, seasons, and visit years in which cases arose. WHIMS[3] is an ancillary study to the hormone therapy CT composed of white women age ≥ 65 at randomization, and free of dementia at baseline. African and Hispanic/Latino ancestry women were obtained from the WHI SNP Health Association Resource (SHARe) project[4], which includes 8,515 self-identified African ancestry women and 3,587 Hispanic/Latino women who provided written informed consent for study participation.

Supplementary Discussion of Suggestive Findings

Several loci that reached the threshold for suggestive significance in trans-ethnic, fixed effects meta-analyses have biologically plausible relationships with ectopy. For example, rs3922844 is intronic to *SCN5A*, a cardiac sodium channel gene associated with Brugada syndrome[5], cardiomyopathy, long QT syndrome type 3[6], PR interval[7], QRS duration, and atrioventricular conduction[8]. This SNP is also a putative enhancer in the fetal heart, left ventricle, and right atrium. SNPs within the *KIF6* locus (such as p.Trp719Arg or rs20455), have been implicated in several cardiometabolic traits, including LDL cholesterol levels[9], type 2 diabetes[10], and coronary heart disease[10]. Genetic variants in *KIF6* were also identified in a GWAS of obesity and its comorbidities[11]. KIF6 is an intracellular molecular motor protein in the kinesin superfamily and is expressed in many tissues, including coronary arteries and vascular cells[12]. Another suggested gene, *TREM2,* has reported roles in the immune response and chronic inflammation[13]. These and other results are available online to enable further investigation into shared genetic mechanisms underlying arrhythmogenesis. Overall, these findings link ectopy to cardiometabolic conditions and inflammation, which are among the mechanisms already proposed as risk factors for SVE and VE in epidemiology studies.

Supplementary Table S1. Genotyping, quality control, imputation & analysis, by cohort and study

Cohort	Genotype			Imputation		
	**Platform**	**Calling Software**	**Filters**	**Software**	**Reference / NCBI Build**	**Reference Panel**
**European ancestry cohorts**						
ARIC	Affymetrix GeneChip SNP Array 6.0	Birdseed	Call rate ≤ 95%<br>HWE $p < 1x10^{-6}$<br>MAF < 1%	MaCH v1.0.16	HapMap 2 Build 36	CEU
CHS	Illumina 370CNV	Illumina BeadStudio	Call rate < 97%, HWE $p < 10^{-5}$, > 2 duplicate errors or Mendelian inconsistencies (for reference CEPH trios), heterozygote frequency = 0, SNP not found in HapMap	BIMBAM v0.99	HapMap 2 Build 36	CEU
MESA	Affymetrix GeneChip SNP Array 6.0	Birdseed v1.33	Call rate ≤ 95%<br>HWE $p < 1x10^{-6}$<br>MAF < 1%	IMPUTE v2	1000G v3 Build 37 3/2012	Phase 1 March 2012 (ALL_1000G_phase1integrated_v3)
WHI-GARNET	Illumina Human Omni1-Quad v1-0 B	BeadStudio v3.1.3.0	Call rate ≤ 98%<br>HWE $p < 1x10^{-4}$	BEAGLE v3.3.1	1000G v3 3/2012	EUR[2]
WHI-MOPMAP	Affymetrix Axiom Genome-Wide Human CEU I	Birdseed	Call rate ≤ 90%<br>HWE $p < 1x10^{-6}$<br>MAF < 0.5%	MaCH minimac	Hapmap 2 Build 36	CEU
WHI-WHIMS	Human OmniExpress Exome-8v1_B Genome-Wide Human	Birdseed	Call rate ≤ 98%<br>HWE $p < 1x10^{-4}$<br>MAF < 1%	MaCH minimac	Hapmap 2 Build 36	CEU
**African ancestry cohorts**						
ARIC	Affymetrix GeneChip SNP Array 6.0	Birdseed	Call rate ≤ 90%<br>MAF < 1%	MaCH v1.0.16	HapMap 2 Build 36	YRI/CEU 1:1
CHS	Illumina	Illumina	Call rate < 97%,	BEAGLE v3.2.1	HapMap 2 and	HapMap 2: CEU, YRI.

	Genotype			Imputation		
Cohort	Platform	Calling Software	Filters	Software	Reference / NCBI Build	Reference Panel
	HumanOmni1-Quad_v1	BeadStudio	HWE p < 10^{-5}, > 2 duplicate errors or Mendelian inconsistencies (for reference CEPH trios), heterozygote frequency = 0, SNP not found in HapMap		HapMap 3 Build 36	HapMap 3: ASW, YRI and CEU. Results were merged across the HapMap 2 and 3 imputations.
MESA	Affymetrix GeneChip SNP Array 6.0	Birdseed v1.33	Call rate ≤ 95%<br>HWE p < $1x10^{-6}$<br>MAF < 1%	IMPUTE v2	1000G v3 Build 37 3/2012	Phase 1 March 2012 (ALL_1000G_phase1integrated_v3)
WHI-SHARe	Affymetrix GeneChip SNP Array 6.0	Birdseed	Call rate ≤ 95%<br>HWE p < $1x10^{-6}$<br>MAF < 1%	MaCH v1.0.16	HapMap 2 Build 36	YRI/CEU 1:1
**Hispanic/Latino ancestry cohorts**						
HCHS/SOL	Illumina HumanOmni2.5-8v1-1 plus custom content[1]	GenomeStudio v2011.1	Call rate ≤ 98%<br>HWE p < $1x10^{-5}$<br>Mendelian errors<br>Duplicate sample discordance	SHAPEIT2 v2.r644<br>IMPUTE2 v2.3.0	1000G v3 3/2012 GRCh37	All ancestries[3]
MESA	Affymetrix GeneChip SNP Array 6.0	Birdseed v1.33	Call rate ≤ 95%<br>HWE p < $1x10^{-6}$<br>MAF < 1%	IMPUTE v2	1000G v3 Build 37 3/2012	Phase 1 March 2012 (ALL_1000G_phase1integrated_v3)
WHI-SHARe	Affymetrix GeneChip SNP Array 6.0	Birdseed	Call rate ≤ 95%<br>HWE p < $1x10^{-6}$<br>MAF < 1%	MaCH v1.0.16	1000G v3 3/2012	All ancestries[3]

Abbreviations: 1000G, 1000 Genomes; ARIC, Atherosclerosis Risk in Communities study; CEU, Northern and Western European ancestry; CHS, Cardiovascular Health Study; EUR, 1000 Genomes European; GARNET, Genomics and Randomized Trials Network; HCHS/SOL, Hispanic Community Health Study/Study of Latinos; HWE, hardy-Weinberg equilibrium; MAF, minor allele frequency; MESA, Multi-Ethnic Study of Atherosclerosis; MOPMAP, Modification of PM-Mediated Arrhythmogenesis in Populations; SHARe, WHI SNP Health Association Resource; SNPs, single nucleotide polymorphisms; WHI, Women's Health Initiative; WHIMS, Women's Health Initiative Memory Study; YRI, Yoruban African ancestry population.

[1] The SOL HCHS Custom 15041502 array (annotation B3, genome build 37) was comprised of 2,427,090 SNPs from a standard Illumina HumanOmni2.5-8v1-1 array and 109,571 custom SNPs

[2] The 1000 Genomes "EUR" continental analysis panel is comprised of CEU (Utah residents with Northern and Western European ancestry), TSI (Toscani in Italia), GBR (British in England and Scotland), FIN (Finnish in Finland), and MXL (Mexican ancestry in Los, Angeles, California) populations.

[3] The 1000 Genomes worldwide reference panel is comprised of four ancestry groups: African (ASW (African Ancestry in Southwest US), LWK (Luhya in Webuye, Kenya), and YRI (Yoruba in Ibadan, Nigeria)), American (CLM (Colombian in Medellin, Colombia), MXL (Mexican ancestry in Los Angeles, California), PUR (Puerto Rican in Puerto Rico)), Asian (CHB (Han Chinese in Beijing, China), CHS (Han Chinese in South China), JPT (Japanese in Tokyo, Japan)), and European (comprised of 5 populations, listed in footnote 2).

Supplementary Table S2. SNPs with suggestive ($5.0 \times 10^{-8} < P < 2.5 \times 10^{-6}$) evidence of association with SVE and VE after fixed-effects meta-analysis of European, African, and Hispanic/Latino ancestry groups

SNP	Genomic region (Build 37)	Gene	Alleles (E/O)	Effect Allele Frequency (E, A, H/L)	OR	95% CI	*P*	N	P_{het}	$Log_{10}BF$	Direction[1]
SVE											
rs3922844	Chr3:38624253	*SCN5A*	T/C	0.31, 0.58, 0.36	1.18	(1.11, 1.26)	1.50×10^{-7}	43,201	0.06	5.57[2]	++-++-++++++-
rs9471077	Chr6:39308742	*KIF6*	A/G	0.62, 0.24, 0.59	0.85	(0.80, 0.91)	7.62×10^{-7}	43,201	0.68	4.60	-------------
rs9827945	Chr3:38845381	---	C/G	0.12, 0.45, 0.12	1.21	(1.12, 1.31)	9.00×10^{-7}	41,711	0.40	4.62	+-+++++++++-?
rs12692501	Chr2:13628448	---	A/G	0.14, 0.36, 0.15	1.20	(1.12, 1.30)	1.23×10^{-6}	41,711	0.94	4.63	++++-+++++++?
rs2234247	Chr6:41131728	*TREM2*	A/G	----, 0.88, 0.97	0.63	(0.52, 0.76)	1.31×10^{-6}	12,697	0.74	4.48	??????-?---??
VE											
rs3787662	Chr21:30524293	*MAP3K7CL*	A/C	0.05, 0.29, 0.14	1.24	(1.13, 1.35)	1.94×10^{-6}	34,152	0.06	4.26	++++?++?+?+-
rs6766673	Chr3:73157288	---	T/C	0.54, 0.53, 0.61	0.88	(0.83, 0.93)	1.94×10^{-6}	42,890	0.04	4.56	+-+-+---++--
rs1722426	Chr2:16907892	---	A/C	0.93, 0.97, 0.79	0.78	(0.71, 0.87)	2.19×10^{-6}	29,364	0.92	4.36	?---?--????-

Abbreviations: A, African ancestry; BF, Bayes' factor; Chr, Chromosome; CI, confidence interval; E, European ancestry; E/O, Effect allele/Other allele; H/L, Hispanic/Latino ancestry; N, number; OR, odds ratio; *P*, p-value; P_{het}, p-value for heterogeneity; SE, standard error; SNP, single nucleotide polymorphism; SVE, supraventricular ectopy; VE, ventricular ectopy.

[1] SVE Direction: 13 populations: ARIC WH – WHI-WHIMS – WHI-MOPMAP controls – WHI-GARNET controls – CHS WH – MESA WH – ARIC BL – WHI-SHARE BW – CHS BL – MESA BL – SOL HIS – WHI-SHARE HIS – MESA HIS; VE Direction: 12 populations: ARIC BL – ARIC WH – SOL HIS – MOPMAP – WHIMS – WHI-SHARE BW – WHI-SHARE HW – WHI-GARNET controls – MESA BL – MESA WH – CHS BL – CHS WH

[2] Posterior probability = 0.35, Direction in MANTRA (E, A, H/L): +++

Supplementary Figure S1. Quantile-quantile plots for each study that contributed to meta-analysis of supraventricular ectopy.

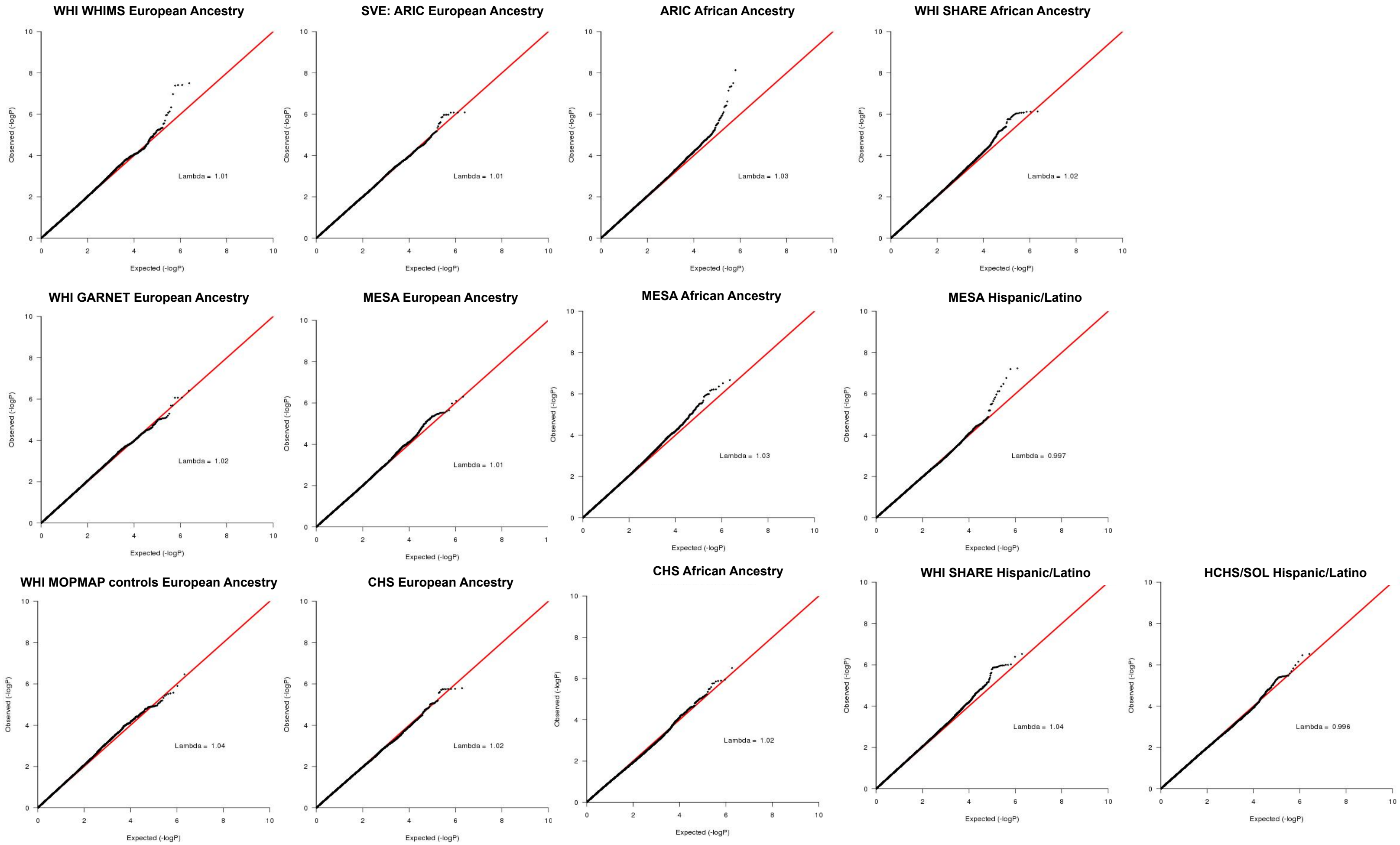

Supplementary Figure S2. Quantile-quantile plots for each study that contributed to meta-analysis of ventricular ectopy.

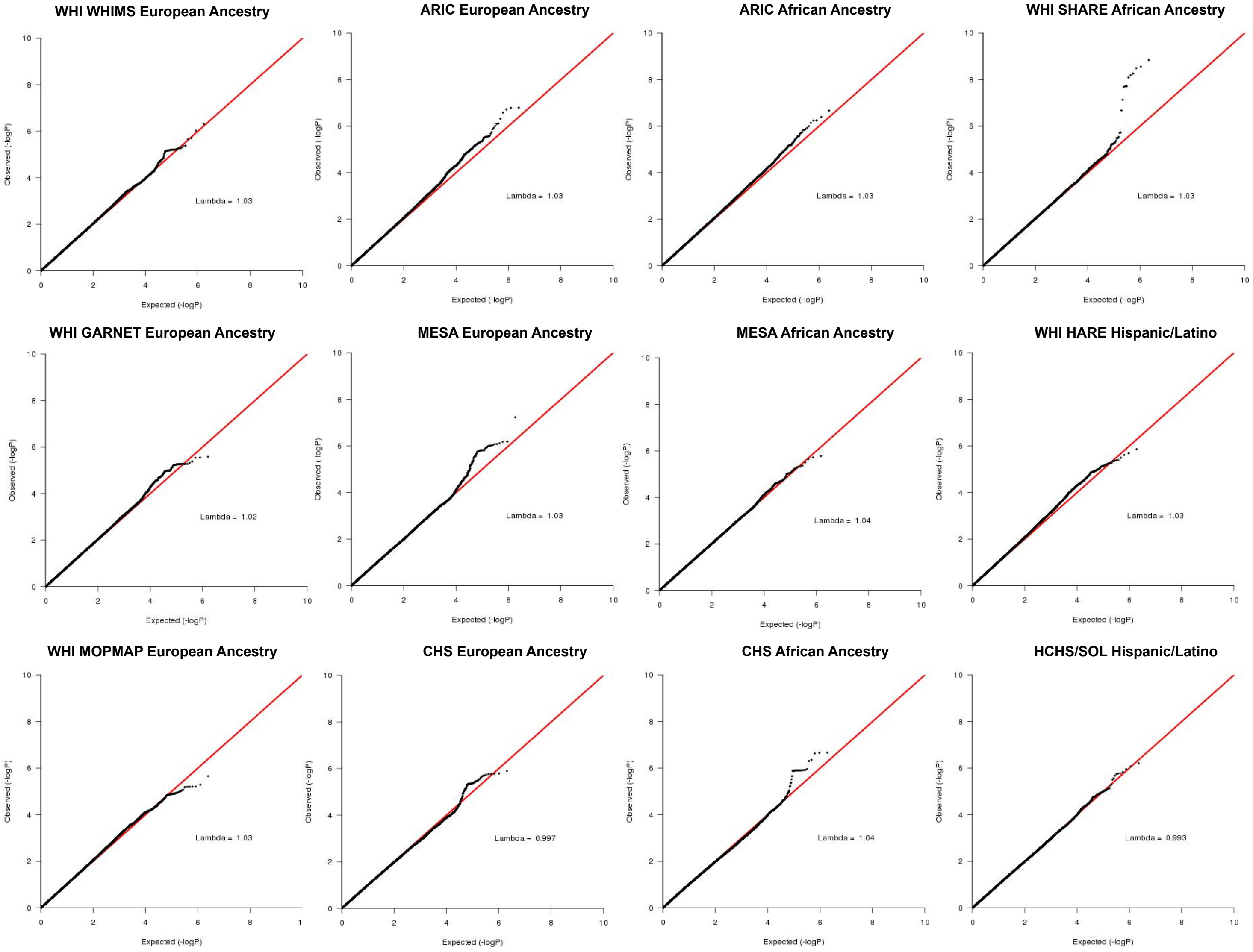

Supplementary Figure S3. Quantile-quantile and Manhattan plots from trans-ethnic, fixed-effects meta-analysis of (A) supraventricular ectopy (SVE) and (B) ventricular ectopy (VE); and from trans-ethnic, multi-trait analysis of (C) SVE & VE.
Dotted horizontal line indicates the 5 x 10^{-8} threshold of genome-wide significance. All plots limited to where ≥ 2 populations (or ancestries in the case of multi-trait analysis) contribute.

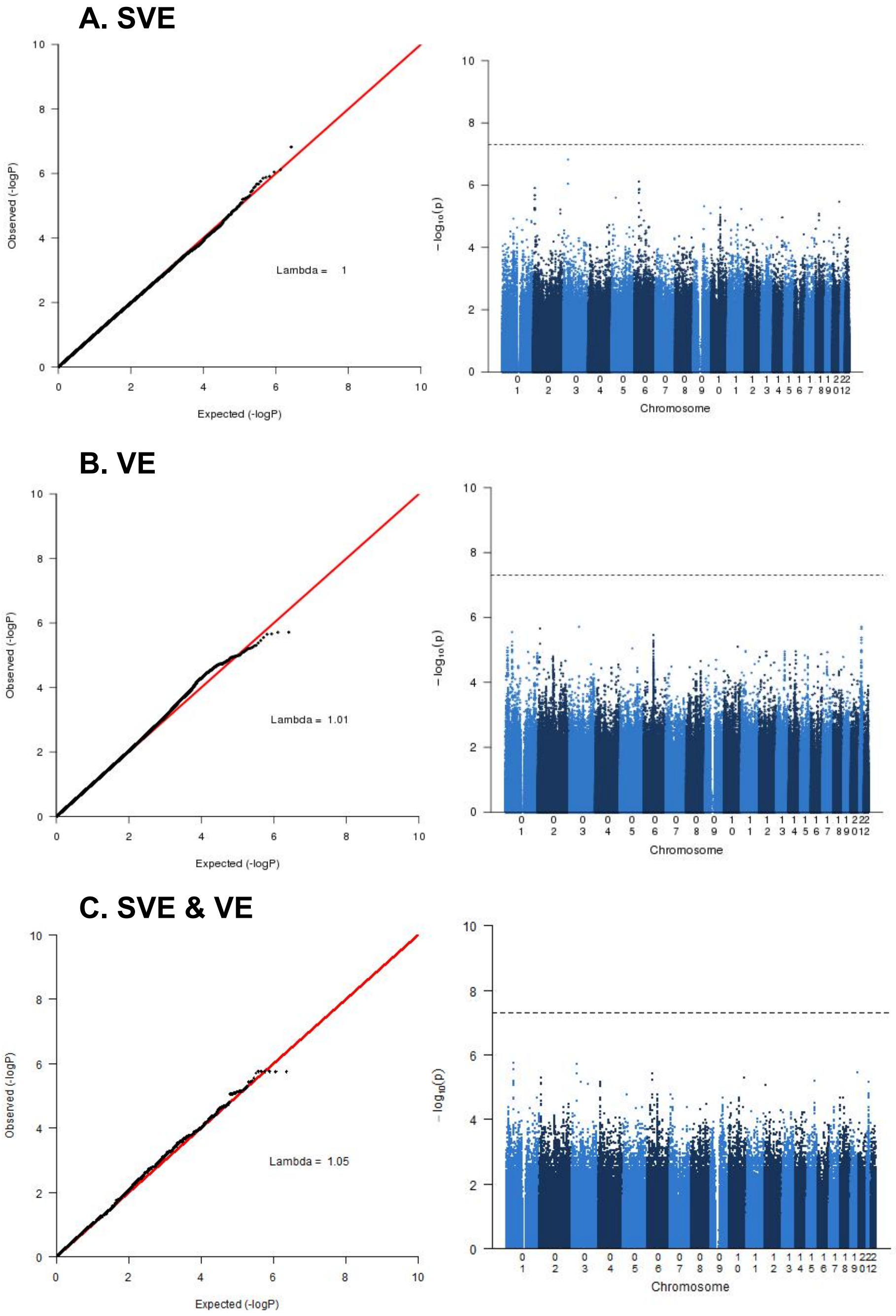

Supplementary Figure S4. Regional plot (A) of rs7545860 from multi-trait analysis of SVE & VE among European ancestry populations, and forest plots of rs7545860 from fixed effects analysis of SVE (B) & VE (C) among European ancestry populations.

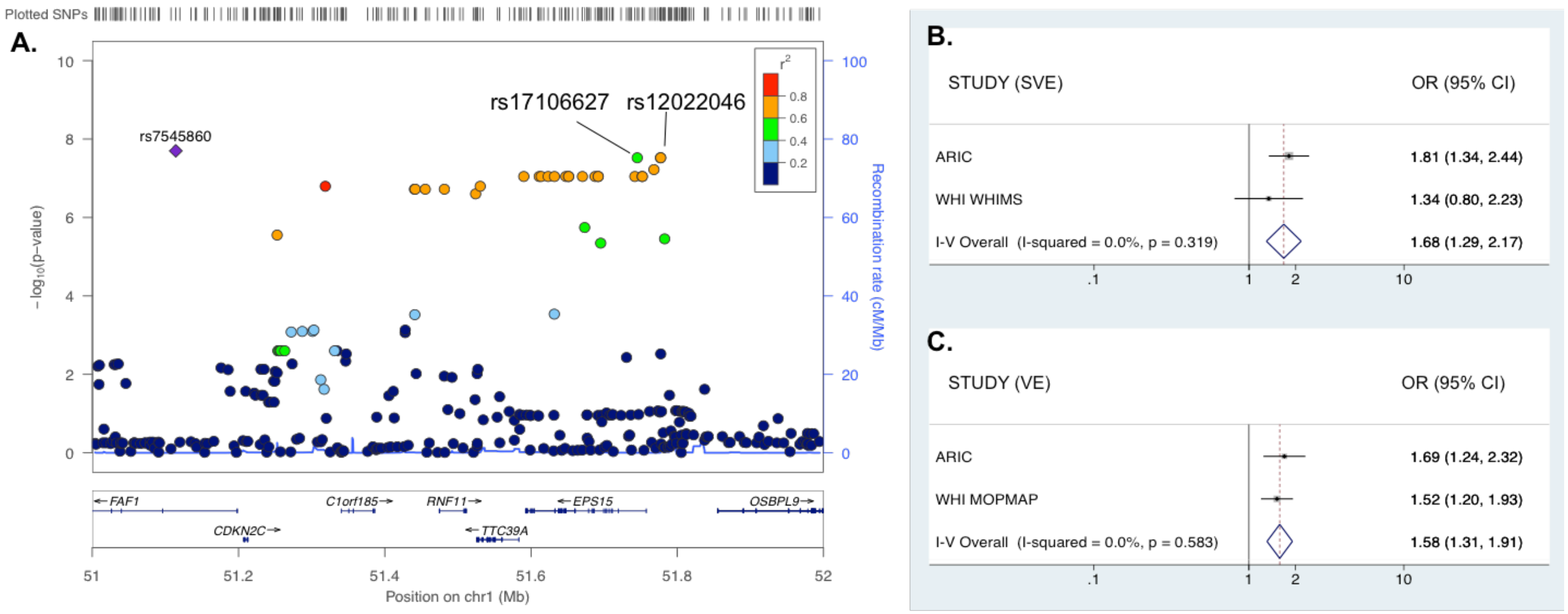

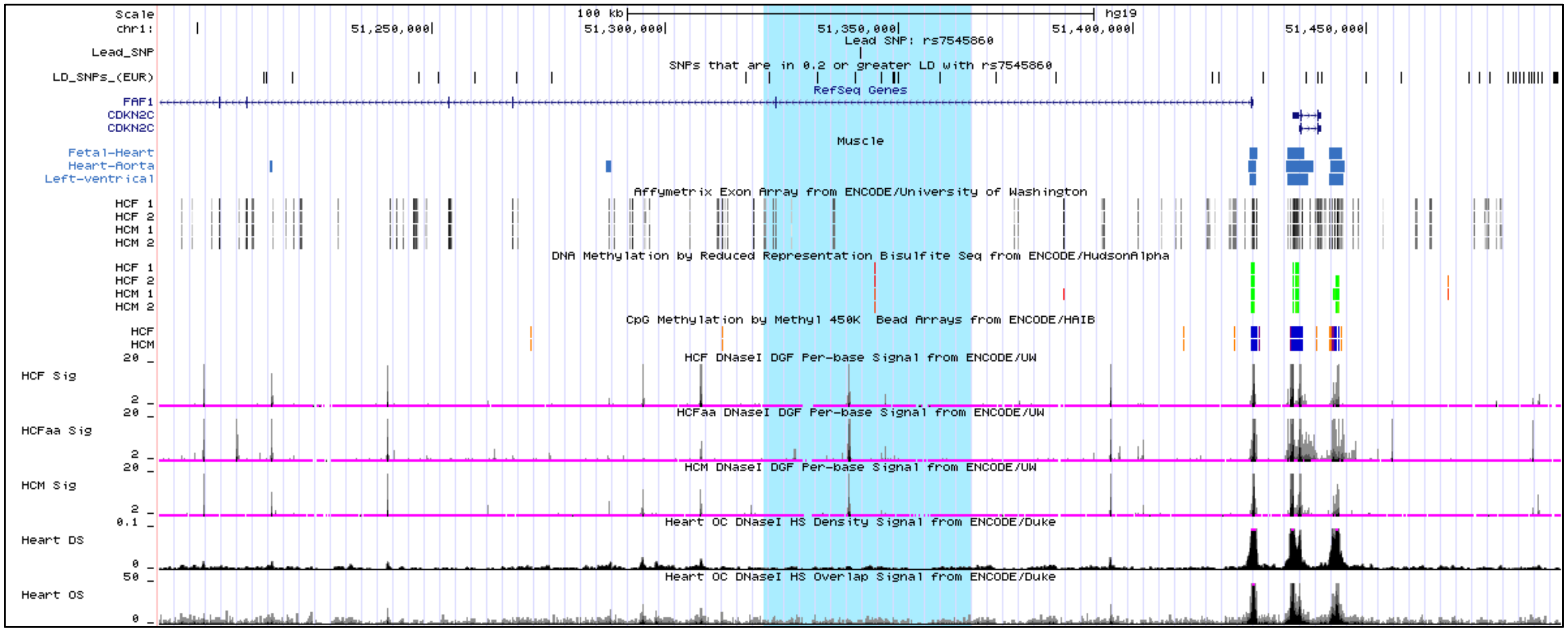

Supplementary Figure S5. UCSC Genome Brower displaying rs7545860 (highlighted), SNPs in linkage disequilibrium (r^2 ≥ 0.2 using EUR reference), and tracks representing DNAse1 hypersensitivity and DNA methylation in cardiac tissues.

Tracks, from top to bottom:

Lead_SNP: SNP with a genome-wide significant association in this study, rs7545860.

LD_SNPs_(EUR): SNPs associated with rs7545860 (linkage disequilibrium > 0.2) using EUR reference, from HaploReg.[14]

Fetal Heart, Heart-Aorta, Left Ventrical: Human fetal heart, heart aorta, and left ventricle DNA methylation by bisulfite sequencing analysis pipeline, MethPipe.[15] Blue intervals indicate hypomethylated regions.

FAF1: Fas-associated factor 1

CDKN2C: Cyclin-dependent kinase inhibitor 2C

HCF & HCM 1/2: Human cardiac fibroblast & myocyte DNA methylation by Affymetrix Exon Array from ENCODE/University of Washington[3] and by reduced representation bisulfite sequencing[16], from ENCODE/HudsonAlpha. Percent of sequenced molecules that are DNA methylated: red (100%), yellow (50%), green (0%).

HCF & HCM: Human cardiac fibroblast & myocyte CpG methylation by Methyl 450K Bead Arrays from ENCODE/HAIB. Methylation status: orange (methylated), purple (partially methylated), bright blue (unmethylated), black (NA).

HCF Sig: Human cardiac fibroblast deoxyribonuclease 1 digital genomic footprinting (DNAse1 DGF) per base signal, from ENCODE/UW.

HCFaa Sig: Human cardiac (adult atrial) fibroblast DNAse1 DGF per base signal, from ENCODE/UW.

HCM Sig: Human cardiac myocyte DNAse1 DGF per-base signal, from ENCODE/UW.

Heart DS/OS: Heart tissue DNAse1 hypersensitivity density / overlap signals, from ENCODE/Duke.

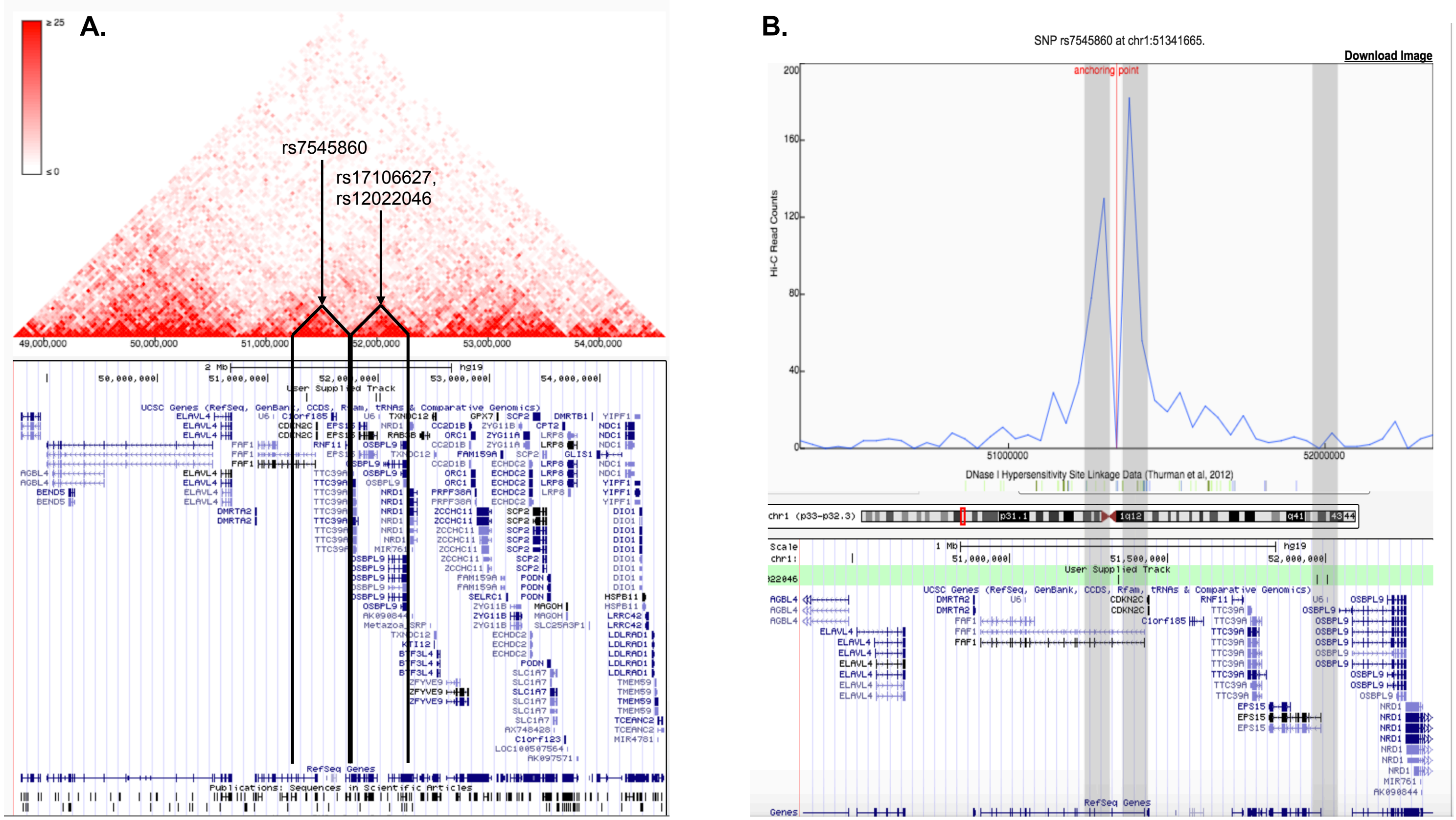

Supplementary Figure S6. (A) Hi-C data on 3D spatial contacts show that rs7545860 and rs17106627, rs12022046 belong to possibly different topological associated domains (TAD).

The rs7545860 variant is proximal in 3D space to *CDKN2C*, *FAF1*, *TTC39A*, and *Corf185*. Variants rs17106627 and rs12022046 are proximal in 3D space to *EPS15*, *RAB3B*, *TTC39A*, *OSBPL9*, *NRD1*, among others. (B) Virtual 4-D figure centered at rs7545860 show that this SNP is in contact with genes *FAF1* and *CDKN2C*, but not in contact with either with rs17106627 or rs1202204.

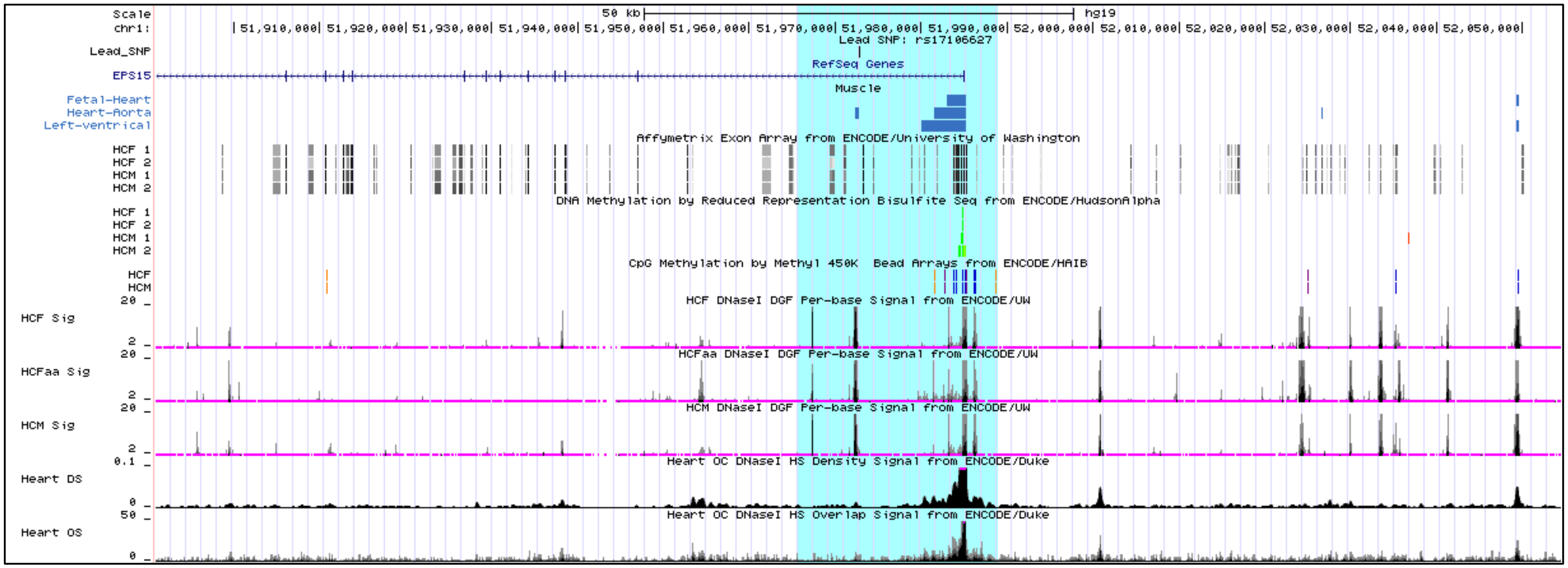

Supplementary Figure S7. UCSC Genome Brower displaying rs17106627 (highlighted) and tracks representing DNAse1 hypersensitivity and DNA methylation in cardiac tissues.

Tracks, from top to bottom:

Lead_SNP: SNP with a genome-wide significant association in this study, rs7545860.

LD_SNPs_(EUR): SNPs associated with rs7545860 (linkage disequilibrium > 0.2) using EUR reference, from HaploReg[14].

Fetal Heart, Heart-Aorta, Left Ventrical: Human fetal heart, heart aorta, and left ventricle DNA methylation by bisulfite sequencing analysis pipeline, MethPipe[15]. Blue intervals indicate hypomethylated regions.

EPS15: Epidermal growth factor receptor pathway substrate 15

HCF & HCM 1/2: Human cardiac fibroblast & myocyte DNA methylation by Affymetrix Exon Array from ENCODE/University of Washington[17] and by reduced representation bisulfite sequencing[16], from ENCODE/HudsonAlpha. Percent of sequenced molecules that are DNA methylated: red (100%), yellow (50%), green (0%).

HCF & HCM: Human cardiac fibroblast & myocyte CpG methylation by Methyl 450K Bead Arrays from ENCODE/HAIB. Methylation status: orange (methylated), purple (partially methylated), bright blue (unmethylated), black (NA).

HCF Sig: Human cardiac fibroblast deoxyribonuclease 1 digital genomic footprinting (DNAse1 DGF) per base signal, from ENCODE/UW.

HCFaa Sig: Human cardiac (adult atrial) fibroblast DNAse1 DGF per base signal, from ENCODE/UW.

HCM Sig: Human cardiac myocyte DNAse1 DGF per-base signal, from ENCODE/UW.

Heart DS/OS: Heart tissue DNAse1 hypersensitivity density / overlap signals, from ENCODE/Duke.

Supplementary Figure S8. Regional (A) and forest plots (B) of the association of rs8086068 and *supraventricular ectopy* in European, African, and Hispanic/Latino ancestry cohorts.

Solid black line is at the null value of 1. Dotted red line signifies the overall point estimate. Study-specific odds ratios (OR) and 95% CIs are denoted by black circles and bars. The subtotal OR estimate across for each ancestry and overall OR estimate is represented by a blue diamond, where diamond width corresponds to 95% CI bounds. The height of the box diamond is inversely proportional to the precision of the OR estimate. The heterogeneity within and among groups is denoted by I-squared.

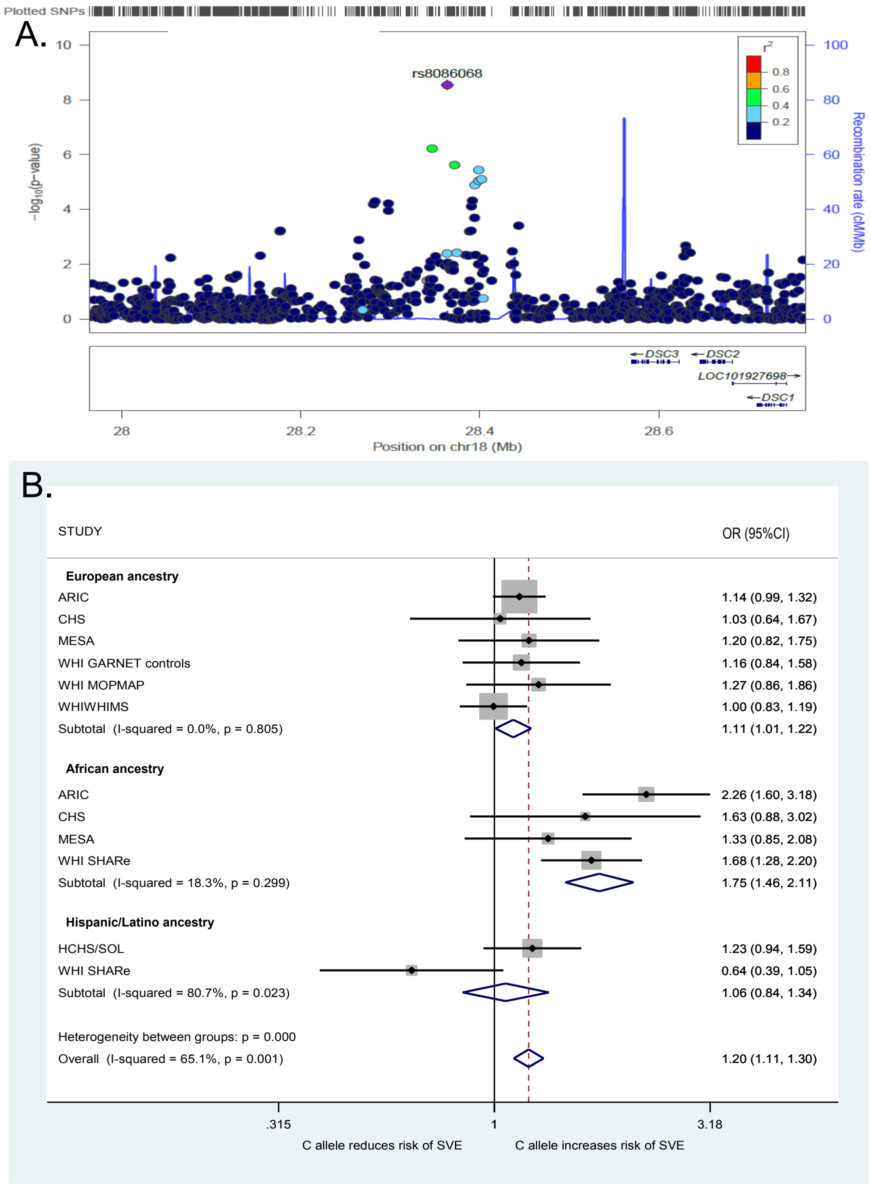

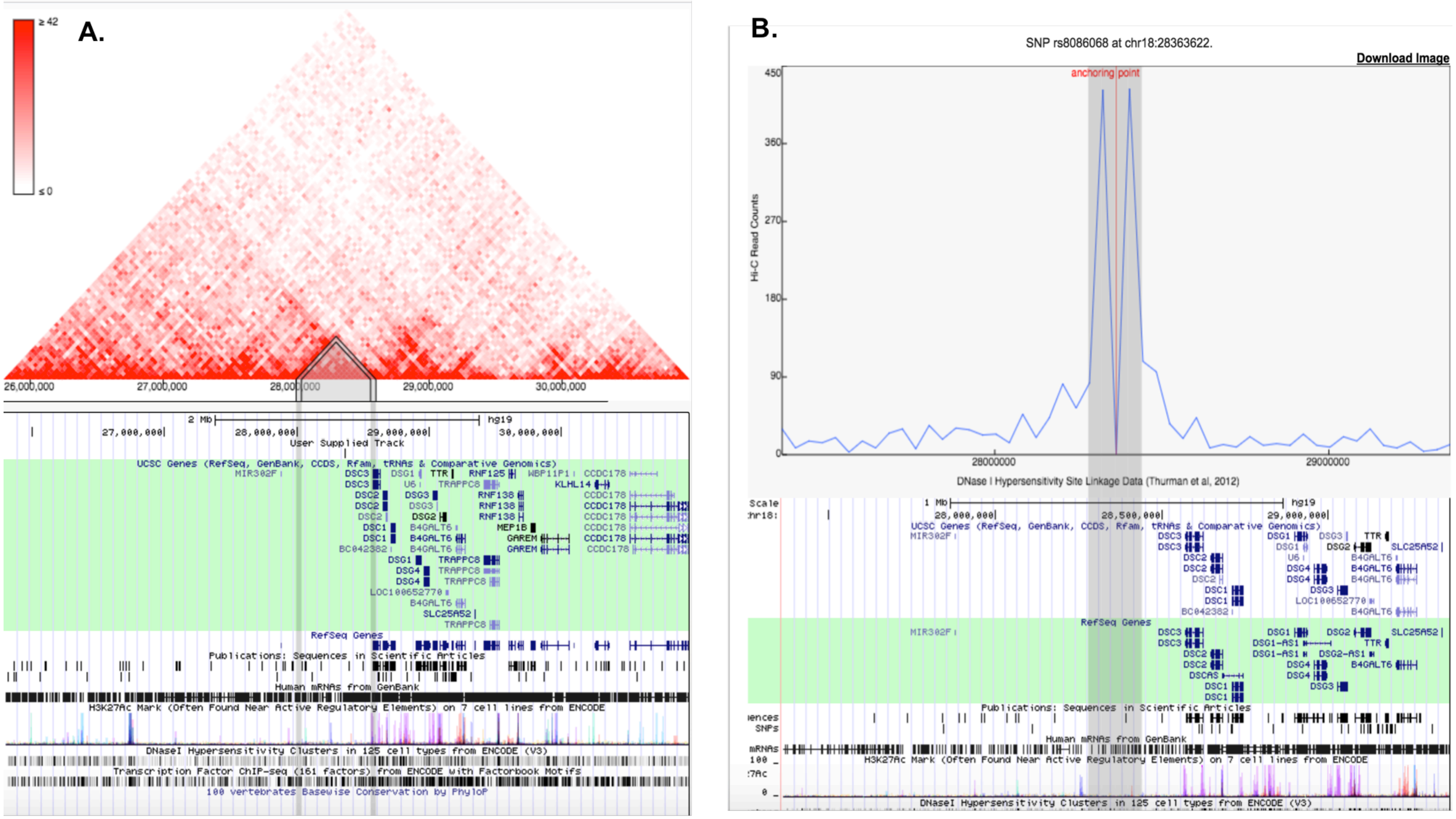

Supplementary Figure S9. (A) Hi-C data on 3D spatial contacts show that rs8086068 may be within the same topological associated domains (TAD) with *DSC3*. (B) In contrast, virtual 4-D figure centered at rs8086068 show little interaction with *DSC3*.

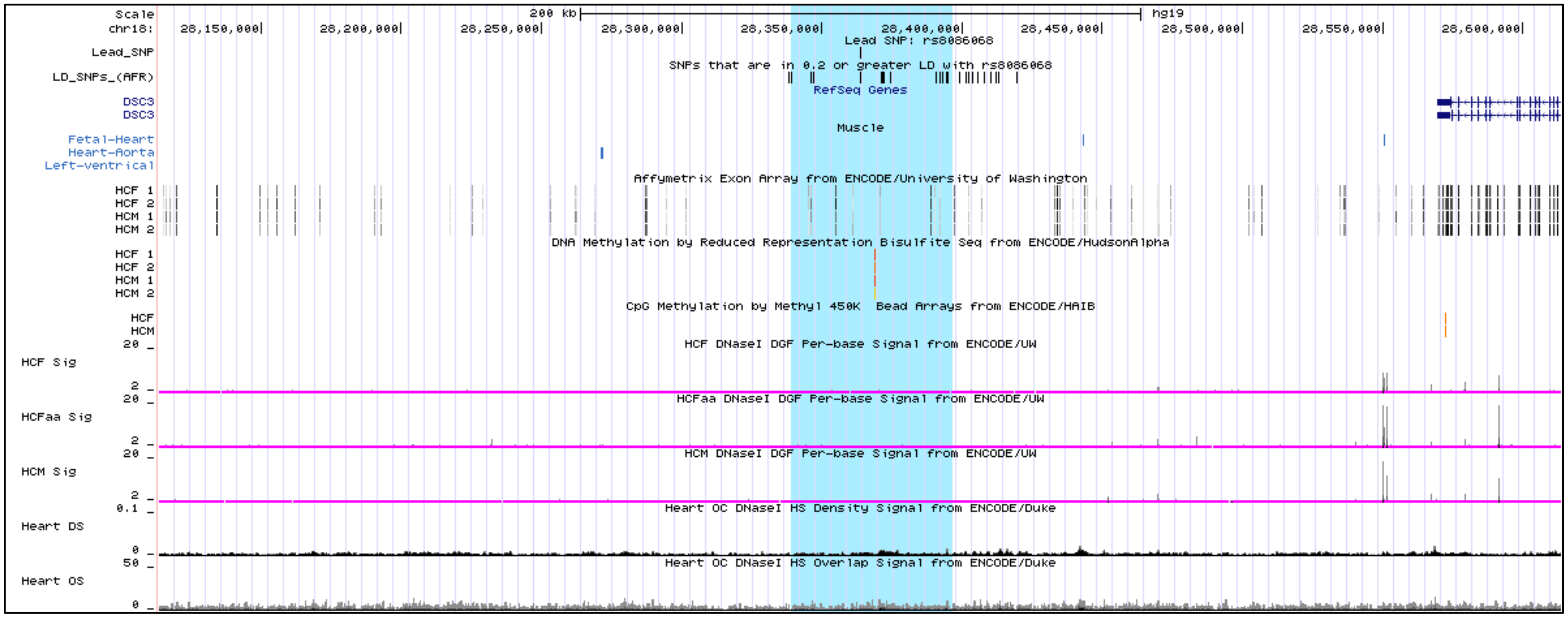

Supplementary Figure S10. UCSC Genome Brower displaying rs8086068 (highlighted), SNPs in linkage disequilibrium ($r^2 \geq 0.2$ using AFR reference), and tracks representing DNAse1 hypersensitivity and DNA methylation in cardiac tissues.

Tracks, from top to bottom:

Lead_SNP: SNP with a genome-wide significant association in this study, rs7545860.

LD_SNPs_(AFR): SNPs associated with rs7545860 (linkage disequilibrium > 0.2) using AFR reference, from HaploReg[14].

Fetal Heart, Heart-Aorta, Left Ventrical: Human fetal heart, heart aorta, and left ventricle DNA methylation by bisulfite sequencing analysis pipeline, MethPipe[15]. Blue intervals indicate hypomethylated regions.

DSC3: Desmocollin 3

HCF & HCM 1/2: Human cardiac fibroblast & myocyte DNA methylation by Affymetrix Exon Array from ENCODE/University of Washington[17] and by reduced representation bisulfite sequencing[16], from ENCODE/HudsonAlpha. Percent of sequenced molecules that are DNA methylated: red (100%), yellow (50%), green (0%).

HCF & HCM: Human cardiac fibroblast & myocyte CpG methylation by Methyl 450K Bead Arrays from ENCODE/HAIB. Methylation status: orange (methylated), purple (partially methylated), bright blue (unmethylated), black (NA).

HCF Sig: Human cardiac fibroblast deoxyribonuclease 1 digital genomic footprinting (DNAse1 DGF) per base signal, from ENCODE/UW.

HCFaa Sig: Human cardiac (adult atrial) fibroblast DNAse1 DGF per base signal, from ENCODE/UW.

HCM Sig: Human cardiac myocyte DNAse1 DGF per-base signal, from ENCODE/UW.

Heart DS/OS: Heart tissue DNAse1 hypersensitivity density / overlap signals, from ENCODE/Duke.

Study-specific acknowledgements and funding sources for participating studies

The Atherosclerosis Risk in Communities Study (ARIC): The Atherosclerosis Risk in Communities Study is carried out as a collaborative study supported by NHLBI contracts (HHSN268201100005C, HHSN268201100006C, HHSN268201100007C, HHSN268201100008C, HHSN268201100009C, HHSN268201100010C, HHSN268201100011C, and HHSN268201100012C), R01HL087641, R01HL59367 and R01HL086694; National Human Genome Research Institute contract U01HG004402; and National Institutes of Health contract HHSN268200625226C. The authors thank the staff and participants of the ARIC study for their important contributions. Infrastructure was partly supported by Grant Number UL1RR025005, a component of the National Institutes of Health and NIH Roadmap for Medical Research.

Cardiovascular Health Study (CHS): This CHS research was supported by NHLBI contracts HHSN268201200036C, HHSN268200800007C, N01HC55222, N01HC85079, N01HC85080, N01HC85081, N01HC85082, N01HC85083, N01HC85086; and NHLBI grants U01HL080295, R01HL087652, R01HL105756, R01HL103612, R01HL120393, R01HL085251 and R01HL130114 with additional contribution from the National Institute of Neurological Disorders and Stroke (NINDS). Additional support was provided through R01AG023629 from the National Institute on Aging (NIA). A full list of principal CHS investigators and institutions can be found at CHS-NHLBI.org. The provision of genotyping data was supported in part by the National Center for Advancing Translational Sciences, CTSI grant UL1TR000124, and the National Institute of Diabetes and Digestive and Kidney Disease Diabetes Research Center (DRC) grant DK063491 to the Southern California Diabetes Endocrinology Research Center. The content is solely the responsibility of the authors and does not necessarily represent the official views of the National Institutes of Health.

Hispanic Community Health Study/Study of Latinos (HCHS/SOL): We thank the participants and staff of the HCHS/SOL study for their contributions to this study. The baseline examination of HCHS/SOL was carried out as a collaborative study supported by contracts from the National Heart, Lung, and Blood Institute (NHLBI) to the University of North Carolina (N01-HC65233), University of Miami (N01-HC65234), Albert Einstein College of Medicine (N01-HC65235), Northwestern University (N01-HC65236), and San Diego State University (N01-HC65237). The following Institutes/Centers/Offices contributed to the first phase of HCHS/SOL through a transfer of funds to the NHLBI: National Institute on Minority Health and Health Disparities, National Institute on Deafness and Other Communication Disorders, National Institute of Dental and Craniofacial Research (NIDCR), National Institute of Diabetes and Digestive and Kidney Diseases, National Institute of Neurological Disorders and Stroke, NIH Institution-Office of Dietary Supplements. The Genetic Analysis Center at the University of Washington was supported by NHLBI and NIDCR contracts (HHSN268201300005C AM03 and MOD03). Genotyping efforts were supported by NHLBI HSN 26220/20054C, NCATS CTSI grant UL1TR000124, and NIDDK Diabetes Research Center (DRC) grant DK063491. This manuscript has been reviewed by the HCHS/SOL Publications Committee for scientific content and consistency of data interpretation with previous HCHS/SOL publications. A complete list of staff and investigators has been provided by

Sorlie P. et al. in Ann Epidemiol. 2010 Aug;20:642-649 and is also available on the study website: http://www.cscc.unc.edu/hchs/.

Multi-Ethnic Study of Atherosclerosis (MESA): This research was supported by contracts HHSN2682015000031, N01-HC-95159, N01-HC-95160, N01-HC-95161, N01-HC-95162, N01-HC-95163, N01-HC-95164, N01-HC-95165, N01-HC-95166, N01-HC-95167, N01-HC-95168, N01-HC-95169 and by grants UL1-TR-000040, UL1-TR-001079, and UL1-RR-025005 from NCRR. Funding for MESA SHARe genotyping was provided by NHLBI Contract N02-HL-6-4278. The provision of genotyping data was supported in part by the National Center for Advancing Translational Sciences, CTSI grant UL1TR000124, and the National Institute of Diabetes and Digestive and Kidney Disease Diabetes Research Center (DRC) grant DK063491 to the Southern California Diabetes Endocrinology Research Center. Further information can be found at:

http://www.ncbi.nlm.nih.gov/projects/gap/cgi-bin/study.cgi?study_id=phs000209.v13.p3

and http://www.mesa-nhlbi.org.

Women's Health Initiative (WHI): The WHI program is funded by the NHLBI, U.S. Department of Health and Human Services through contracts HHSN268201100046C, HHSN268201100001C, HHSN268201100002C, HHSN268201100003C, HHSN268201100004C, and HHSN271201100004C. All contributors to WHI science are listed @ https://www.whi.org/researchers/Documents%20%20Write%20a%20Paper/WHI%20Investigator%20Long%20List.pdf. Within the WHI, The SNP Health Association Resource project (WHI-SHARe) was funded by the National Heart, Lung and Blood Institute, National Institutes of Health, U.S. Department of Health and Human Services through contract N02HL64278 (Kooperberg). The Women's Health Initiative Memory Study (WHI-WHIMS) was funded by the National Institutes of Health and supported through funding by Wyeth-Ayerst laboratories. A GWAS of Hormone Treatment and CVD and Metabolic Outcomes within the Genomics and Randomized Trials Network (WHI-GARNET) was funded by the National Human Genome Research Institute, National Institutes of Health, U.S. Department of Health and Human Services through cooperative agreement U01HG005152 (Reiner). All contributors to GARNET science are listed @ https://www.garnetstudy.org/Home. The Modification of PM-Mediated Arrhythmogenesis in Populations (WHI-MOPMAP) was funded by the National Institute of Environmental Health Sciences, National Institutes of Health, U.S. Department of Health and Human Services through grant R01ES017794 (Whitsel) and the University of North Carolina Cancer Research Fund. Support also was provided by the National Human Genome Research Institute R01HG006292 and R01HG006703 (YL) and National Heart, Lung, and Blood Institute R01HL129132 (YL, APR).

References

1. Genomics and Randomized Trials Network (GARNET). https://www.genome.gov/27541119/ (2013).

2. National Institutes of Environmental Health Sciences. Modification of PM-mediated arrhythmogenesis in populations. http://projectreporter.nih.gov/project_info_description.cfm?aid=7984809&icde=19283008.

3. Shumaker, S. A. *et al.* The Women's Health Initiative Memory Study (WHIMS): a trial of the effect of estrogen therapy in preventing and slowing the progression of dementia. *Control Clin Trials* **19,** 604-621 (1998).

4. SHARe: SNP Health Association Resource Project. https://www.nhlbi.nih.gov/research/resources/genetics-genomics/share (2011).

5. Bezzina, C. R. *et al.* Common variants at SCN5A-SCN10A and HEY2 are associated with Brugada syndrome, a rare disease with high risk of sudden cardiac death. *Nat Genet* **45,** 1044-1049 (2013).

6. Newton-Cheh, C. *et al.* Common variants at ten loci influence QT interval duration in the QTGEN Study. *Nat Genet* **41,** 399-406 (2009).

7. Butler, A. M. *et al.* Novel loci associated with PR interval in a genome-wide association study of 10 African American cohorts. *Circ Cardiovasc Genet* **5,** 639-646 (2012).

8. Sotoodehnia, N. *et al.* Common variants in 22 loci are associated with QRS duration and cardiac ventricular conduction. *Nature genetics* **42,** 1068-1076 (2010).

9. Ference, B. A., Yoo, W., Flack, J. M. & Clarke, M. A common KIF6 polymorphism increases vulnerability to low-density lipoprotein cholesterol: two meta-analyses and a meta-regression analysis. *PLoS One* **6,** e28834 (2011).

10. Wu, G., Li, G. B., Dai, B. & Zhang, D. Q. Novel KIF6 polymorphism increases susceptibility to type 2 diabetes mellitus and coronary heart disease in Han Chinese men. *J Diabetes Res* **2014,** 871439 (2014).

11. Comuzzie, A. G. *et al.* Novel genetic loci identified for the pathophysiology of childhood obesity in the Hispanic population. *PLoS One* **7,** e51954 (2012).

12. Shiffman, D. *et al.* Effect of pravastatin therapy on coronary events in carriers of the KIF6 719Arg allele from the cholesterol and recurrent events trial. *Am J Cardiol* **105,** 1300-1305 (2010).

13. Ford, J. W. & McVicar, D. W. TREM and TREM-like receptors in inflammation and disease. *Curr Opin Immunol* **21,** 38-46 (2009).

14. Ward, L. D. & Kellis, M. HaploReg: a resource for exploring chromatin states, conservation, and regulatory motif alterations within sets of genetically linked variants. *Nucleic Acids Res* **40,** D930-D934 (2012).

15. Song, Q. *et al.* A reference methylome database and analysis pipeline to facilitate integrative and comparative epigenomics. *PLoS One* **8,** e81148 (2013).

16. Cokus, S. J. *et al.* Shotgun bisulphite sequencing of the Arabidopsis genome reveals DNA methylation patterning. *Nature* **452,** 215-219 (2008).

17. Hansen, R. S. *et al.* Sequencing newly replicated DNA reveals widespread plasticity in human replication timing. *Proc Natl Acad Sci U S A* **107,** 139-144 (2010).
